# Relationships Among Periodontal Awareness, Clinical Findings, and Oral Health-Related Quality of Life in Turkish Cypriot Adults

**DOI:** 10.3390/healthcare14162591

**Published:** 2026-08-18

**Authors:** Bektaş Cangil, Özge Uzuner Bilgiç, Halil İbrahim Taşer

**Affiliations:** Department of Periodontology, Faculty of Dentistry, Cyprus Health and Social Sciences University, Morphou 99750, Cyprus; ozge.uzunerbilgic@kstu.edu.tr (Ö.U.B.); halil.taser@kstu.edu.tr (H.İ.T.)

**Keywords:** periodontal disease, awareness, quality of life, OHIP-14, gingival index, plaque index, Ramfjord periodontal index, bleeding on probing, pocket depth, dental scaling

## Abstract

**Background/Objectives:** The primary aim of this observational study was to evaluate the relationships among periodontal awareness, oral health behaviors, self-reported periodontal symptoms, objective clinical periodontal findings, and oral health-related quality of life in Turkish Cypriot adults aged 30 years and older living in Northern Cyprus, and to investigate potential discrepancies between self-reported indicators and objective clinical findings. A three-week clinical re-evaluation performed after professional tooth cleaning and standardized oral hygiene instruction was included as a secondary and descriptive component of the study. **Materials and Methods:** A total of 423 adults completed a 31-item face-to-face questionnaire assessing sociodemographic characteristics, periodontal awareness, oral health behaviors, and OHIP-14. GI, PI, BOP, and RPI were recorded at baseline and three weeks later. Participants were classified as periodontal health, gingivitis-compatible, or periodontitis-compatible. Non-parametric tests were applied (*p* < 0.05). **Results:** GI, PI, BOP, and RPI decreased significantly at T3 (*p* < 0.001). Gingival bleeding, swelling, and halitosis were associated with clinical indices (*p* < 0.001). However, periodontal awareness indicators and oral health behaviors were not consistently associated with clinical periodontal status, indicating incomplete concordance between self-reported awareness and objective clinical findings. Women reported more regular dental visits, previous scaling, and adjunctive product use. Higher education was associated with greater awareness and preventive behaviors. OHIP-14 associations with clinical parameters were weak. **Conclusions:** Overall, significant short-term reductions in clinical periodontal indices were observed after standardized professional tooth cleaning and oral hygiene instruction; however, awareness, behaviors, clinical findings, and OHRQoL did not demonstrate consistent concordance across all comparisons. These findings support professional follow-up and objective periodontal evaluation.

## 1. Introduction

Periodontal diseases are among the most common chronic inflammatory diseases affecting the adult population worldwide and present across a broad clinical spectrum ranging from gingival inflammation to progressive destruction of periodontal tissues. The World Health Organization (WHO) defines periodontal diseases as major public health problems affecting global oral health [1]. Periodontal diseases not only cause loss of tooth-supporting tissues but may also adversely affect individuals’ quality of life through pain, functional impairment, esthetic concerns, and difficulties in social interactions [2,3]. In addition, periodontal inflammation has been reported to show bidirectional relationships with systemic health and may be associated with cardiovascular diseases, diabetes mellitus, and other chronic inflammatory conditions [4]. Periodontal diseases have been shown to extend beyond the boundaries of oral tissues and to create an inflammatory burden that affects systemic health, influences multiple physiological systems, and reduces the overall quality of life of affected individuals [5]. Therefore, increasing awareness of periodontal diseases is a critical step for improving both oral health and overall quality of life.

Individual awareness, preventive oral health behaviors, and regular professional care are considered important components in the prevention of periodontal diseases. Nevertheless, the relationship between individuals’ level of knowledge about periodontal diseases, symptom perception, and oral health behaviors and their objective clinical periodontal status has been shown not to be consistently aligned [6,7]. Some individuals may be unable to correctly identify signs of periodontal disease, whereas others may have high levels of awareness but still present with clinically unfavorable periodontal findings. Similarly, clinical signs of periodontal disease may also be observed in individuals who report regular oral care behaviors.

In recent years, increasing attention has been paid to possible discrepancies between individuals’ perceptions of their periodontal health and clinically determined periodontal status. This situation points to the concept of a “self-awareness/clinical findings gap,” which describes the gap between patients’ periodontal awareness and objective clinical findings. This discrepancy demonstrates that the way individuals assess their periodontal health may not always reflect clinical reality and highlights the limitations of approaches based solely on subjective assessments [7,8].

Similarly, the relationship between oral health-related quality of life (OHRQoL) and clinical periodontal status has also been reported to be complex. Some individuals with clinically evident periodontal findings report limited impairment in quality of life, whereas some individuals with milder clinical changes may experience marked deterioration in oral health-related quality of life [9,10,11]. This suggests that the impact of periodontal diseases on individuals cannot be explained by clinical indicators alone and that psychological, behavioral, and sociodemographic factors should also be considered. Although OHIP-14 is not a periodontal disease-specific instrument, it was selected in this study to evaluate the functional, physical, psychological, and social effects of oral health within a common framework among individuals with different clinical periodontal statuses. Because the study included not only individuals with findings compatible with periodontitis but also those with clinical periodontal health and findings compatible with gingivitis, a generic OHRQoL measure enabled comparisons across the different periodontal status categories. Moreover, the demonstrated validity and reliability of the Turkish OHIP-14 supported its use in this study population [12].

Previous studies have shown that individuals’ self-assessments of their periodontal health do not always correspond with objective clinical findings and that the relationship between clinical periodontal status and oral health-related quality of life may vary among individuals [13,14]. However, the interrelationships among periodontal awareness, self-reported periodontal symptoms, oral health behaviors, objective clinical periodontal findings, and oral health-related quality of life within the same adult sample remain insufficiently clarified. To the best of our knowledge, data comprehensively evaluating these variables within the same study framework among Turkish Cypriot adults living in Northern Cyprus are limited. Consequently, it remains unclear whether individuals with greater periodontal awareness or more favorable oral health behaviors exhibit better clinical periodontal findings, to what extent self-reported periodontal symptoms correspond with objective clinical indices, and whether oral health-related quality of life differs according to clinical periodontal status. Data evaluating short-term clinical periodontal changes after standardized professional tooth cleaning and oral hygiene instruction in the same study population characterized by these variables are also limited. Clarifying these relationships is important for planning community-based preventive periodontal programs because self-reported assessments of knowledge, behaviors, or symptoms alone may be insufficient to identify individuals who require professional periodontal examination.

The primary aim of this study was to evaluate the relationships among periodontal awareness, self-reported oral health behaviors and periodontal symptoms, objective clinical periodontal findings, and oral health-related quality of life in Turkish Cypriot adults aged 30 years and older. The study also aimed to identify areas of concordance and discordance between individuals’ self-assessments of their periodontal health and their clinically measured periodontal status. The secondary aim was to examine short-term changes in clinical periodontal parameters three weeks after professional tooth cleaning and oral hygiene instruction. Accordingly, rather than establishing causal relationships, the study provides an integrated evaluation of periodontal awareness, oral health behaviors, periodontal symptoms, quality of life, clinical findings, and short-term clinical response within the same study population.

## 2. Materials and Methods

### 2.1. Study Design and Participants

This observational study was conducted among Turkish Cypriot adults aged 30 years and older living in Northern Cyprus and included a short-term pre–post clinical follow-up component. The lower age limit of 30 years was selected not as a diagnostic threshold for periodontal disease, but as a prespecified eligibility criterion defining the adult target population of the study. Including adults aged 30 years and older enabled periodontal awareness and clinical findings to be evaluated in a population with longer cumulative exposure to periodontal risk factors and a greater likelihood of clinically meaningful periodontal changes. This age threshold is also consistent with internationally recognized periodontal epidemiological surveillance conventions: the Centers for Disease Control and Prevention/American Academy of Periodontology (CDC/AAP) periodontitis surveillance framework restricts prevalence estimation to dentate adults aged 30 years and older, reflecting the markedly higher burden of periodontal attachment loss and probing depth observed from the fourth decade of life onward [15]. Adopting the same lower age boundary therefore allows the present findings to be situated within, and compared against, this widely used reference population. In addition, because the target population comprised 204,007 adults aged 30 years and older living in Northern Cyprus, consistency was maintained among the eligibility criterion, target population definition, and sampling frame. Based on this prespecified target population, the minimum required sample size was calculated as 384 participants using a 95% confidence level, a 5% margin of error, and an expected prevalence of 50%. To account for potential non-participation and missing data, 500 individuals were invited to participate. Of those invited, 459 agreed to participate and attended the clinical examination. After the prespecified eligibility and exclusion criteria had been applied, 423 participants were included in the final analysis. The final analytical participation rate was calculated as 84.6%. Exclusion criteria were pregnancy, the presence of advanced systemic disease, periodontal treatment within the previous three months, antibiotic or anti-inflammatory drug use, the presence of acute oral infection, and missing data. The study was conducted in accordance with the principles of the Declaration of Helsinki and was approved by the Scientific Research Ethics Committee of Cyprus Health and Social Sciences University (Approval No: KSTU/2025/038). Written informed consent was obtained from all participants before participation.

### 2.2. Questionnaire Assessment

Data were collected between August and December 2025 using a structured questionnaire administered face-to-face. The questionnaire consisted of 31 items and included sociodemographic characteristics, periodontal awareness, oral health behaviors, and the Turkish version of the Oral Health Impact Profile-14 (OHIP-14), whose validity and reliability have been demonstrated [12]. OHIP-14 was selected because the study population comprised individuals with different clinical periodontal statuses rather than only those with clinical findings compatible with periodontitis. Use of the scale enabled the functional, physical, psychological, and social effects of oral health to be assessed within a common generic OHRQoL framework and allowed comparison of the study-defined clinical periodontal status categories. In accordance with the prespecified study protocol, the complete 31-item questionnaire covering sociodemographic characteristics, periodontal awareness, oral health behaviors, and OHIP-14 was administered only at baseline. The questionnaire was designed to evaluate cross-sectional relationships among self-reported characteristics, periodontal awareness, oral health behaviors, oral health-related quality of life, and baseline clinical periodontal findings. Because changes in self-reported awareness, behaviors, symptoms, or oral health-related quality of life were not planned as follow-up outcomes, no part of the questionnaire was readministered at the three-week follow-up. Only selected clinical periodontal parameters were repeated at the three-week re-evaluation.

In addition, readministering OHIP-14 after only three weeks was considered likely to create temporal overlap between experiences reported at baseline and follow-up and to limit independent interpretation of short-term changes in OHRQoL; therefore, repeat administration of the scale was not included in the prespecified study design.

### 2.3. Clinical Periodontal Assessment

Clinical periodontal examinations were performed by a single researcher under standardized conditions using a UNC-15 periodontal probe (EASMAR Industries, Sialkot, Pakistan). The gingival index (GI), plaque index (PI), bleeding-on-probing index (BOP), and Ramfjord periodontal index (RPI) were recorded. Periodontal pocket depth measurements were performed on Ramfjord teeth.

Baseline assessments were defined as T0, and follow-up assessments were defined as T3.

These parameters were selected to assess complementary aspects of periodontal status. PI was used to assess supragingival plaque accumulation, whereas GI and BOP were used to evaluate gingival inflammation and the inflammatory response of the periodontal tissues. Within the epidemiological framework of the study, RPI provided a standardized summary assessment of periodontal involvement. Periodontal probing depth (PPD) measurements at the Ramfjord teeth were recorded to evaluate periodontal pocket findings and site-specific changes. Collectively, these measurements enabled assessment of plaque accumulation, gingival inflammation, bleeding response, and broader periodontal involvement.

### 2.4. Clinical Periodontal Status Categories

Participants were categorized according to baseline clinical examination findings as having clinical periodontal health, a clinical condition consistent with gingivitis, or a clinical condition consistent with periodontitis.

Clinical periodontal health was defined as the absence of clinical inflammation and bleeding on probing [16]. A clinical condition consistent with gingivitis was defined as the presence of gingival inflammation and/or bleeding on probing without increased periodontal pocket depth suggestive of periodontal tissue destruction.

A clinical condition consistent with periodontitis was defined as the presence of periodontal pocket depth (PPD) ≥ 5 mm at least at one Ramfjord tooth site, accompanied by bleeding on probing and/or clinical inflammatory findings.

Because full-mouth clinical attachment level measurements and standardized radiographic bone-loss assessments were not performed, these categories represent study-specific clinical periodontal status categories rather than definitive periodontal diagnoses according to the 2017 World Workshop Classification of Periodontal and Peri-Implant Diseases and Conditions. Therefore, cases consistent with periodontitis were not further classified by stage or grade.

### 2.5. Standardized Professional Tooth Cleaning, Oral Hygiene Instruction, and Follow-Up

Following the T0 assessments, all participants received a standardized professional care protocol comprising professional tooth cleaning and standardized oral hygiene instruction. Clinically detectable plaque and calculus deposits, when present, were removed using ultrasonic instruments (Woodpecker, Guilin Woodpecker Medical Instrument Co., Ltd., Guilin, China), with supplementary hand instrumentation using periodontal scalers when clinically required. To standardize the care provided after baseline and ensure comparability of the three-week clinical assessments, the same protocol was applied to all study-defined clinical periodontal status categories. In participants without clinical evidence of periodontitis, the procedure was performed as preventive professional care rather than as active periodontitis treatment. This approach reflected the established principle that primary prevention of periodontitis relies on professional plaque and calculus removal combined with reinforced oral hygiene instruction, irrespective of whether periodontitis is already clinically evident [6]. Applying the same standardized protocol across all study-defined clinical periodontal status categories also ensured that the three-week within-participant comparisons were not confounded by differential care exposure between groups, and reflected routine preventive dental practice, in which professional prophylaxis is offered independently of periodontitis status. No adjunctive antibiotic or anti-inflammatory therapy was administered. The same clinical periodontal parameters were reassessed three weeks later (T3).

After the procedure, all participants received standardized oral hygiene instruction. At the three-week follow-up (T3), only selected clinical periodontal parameters were reassessed; the 31-item questionnaire was not readministered.

The three-week clinical re-evaluation constituted a secondary and descriptive component of the study and was performed to characterize early within-participant changes in selected clinical periodontal parameters rather than to reconfirm the established effectiveness of professional tooth cleaning.

The obtained data were coded and transferred to a digital database. Missing and erroneous records were cleaned, and all analyses were performed using anonymous patient codes.

### 2.6. Statistical Analysis Methods

Descriptive statistics were used in the analysis of the data. Continuous variables were summarized using mean, standard deviation, median, minimum, and maximum values.

For continuous variables that did not show normal distribution, the Mann–Whitney U test was used for comparisons between two groups, and the Kruskal–Wallis test was used for comparisons among more than two groups. When the Kruskal–Wallis test indicated a statistically significant difference, post hoc pairwise comparisons with Bonferroni adjustment were performed to identify the groups responsible for the difference. Paired comparisons between T0 and T3 measurements were evaluated using the Wilcoxon signed-rank test.

Categorical variables were compared using Fisher’s exact test. Relationships between continuous variables were analyzed using Spearman’s rank correlation coefficient.

The level of statistical significance was accepted as *p* < 0.05. All analyses were performed using MedCalc Statistical Software version 12.7.7 (MedCalc Software bvba, Ostend, Belgium).

## 3. Results

### 3.1. Participant Characteristics

A total of 423 participants were included in the study. Women constituted 68.3% of the participants (*n* = 289), and men constituted 31.7% (*n* = 134). The majority of participants were in the 30–45-year age group (64.8%), and 48.7% held a bachelor’s degree. Overall, 43.8% of participants reported regular dental visits, 74.9% reported having previously undergone professional dental scaling, and 35.7% reported using adjunctive oral hygiene products. In the clinical assessment, 48.2% of participants were categorized as having clinical periodontal health, 42.3% as having a clinical condition consistent with gingivitis, and 9.5% as having a clinical condition consistent with periodontitis (Table 1).

### 3.2. Clinical Changes After Standardized Professional Tooth Cleaning and Oral Hygiene Instruction

The three-week clinical re-evaluation was completed by all participants, and paired T0–T3 analyses were performed for all 423 participants.

At the three-week follow-up after the standardized professional tooth cleaning and oral hygiene instruction protocol, GI, bleeding on probing percentage (BOP), PI, and RPI values were significantly lower than their respective baseline values (*p* < 0.001 for all comparisons; Table 2).

In site-specific analyses of the Ramfjord teeth, significant reductions in PPD were observed on the distobuccal, mesiobuccal, distopalatal, and mesiopalatal surfaces of tooth 16; the distolabial, mesiolabial, distopalatal, and mesiopalatal surfaces of tooth 21; the mesiobuccal, distopalatal, and mesiopalatal surfaces of tooth 24; the distobuccal, mid-buccal, mesiobuccal, distolingual, and mesiolingual surfaces of tooth 36; and the distobuccal, mesiobuccal, distolingual, and mesiolingual surfaces of tooth 44. No statistically significant change in PPD was identified on any surface of tooth 41. Detailed site-specific values and *p*-values are presented in Supplementary Table S1.

### 3.3. Gender

Sex-based differences were identified in several self-reported symptoms, preventive oral health behaviors, and periodontal awareness indicators. Pain was reported more frequently by women than by men (56.4% vs. 45.5%; *p* = 0.046). The distribution of responses regarding gingival bleeding during toothbrushing also differed significantly by sex (*p* = 0.015); men more frequently responded “yes,” whereas women more frequently responded “sometimes.”

Regular dental visits (48.3% vs. 34.3%; *p* = 0.008), previous professional dental scaling (78.1% vs. 68.7%; *p* = 0.040), and use of dental floss or an interdental brush (39.9% vs. 27.1%; *p* = 0.012) were reported more frequently by women. Women also demonstrated greater awareness of the preventive role of professional dental scaling (*p* = 0.039) and of the association between periodontal diseases and cardiovascular disease or diabetes mellitus (*p* = 0.035; Table 3).

When OHIP-14 items were evaluated, a significant sex-based difference was identified only for the OHIP-4 item score, which was higher among women than men (*p* = 0.038). No statistically significant sex-based differences were observed for the other OHIP-14 items.

### 3.4. Age

Significant differences were detected among age groups in some periodontal awareness and behavioral variables (*p* < 0.05). The rate of considering gum disease as an infection was higher in the >60-year age group, whereas smoking was more common in younger and middle-aged groups. In addition, the distribution of responses regarding gingival bleeding during toothbrushing differed significantly among age groups (*p* = 0.041; Table 3).

With respect to OHIP items, statistically significant differences according to age group were identified in the OHIP-1, OHIP-3, OHIP-4, OHIP-6, and OHIP-9 items (*p* < 0.05). OHIP-1 item scores increased with age, whereas OHIP-3 and OHIP-4 item scores were higher particularly in the 50–60-year age group. OHIP-6 item scores also increased with age and were higher among individuals older than 50 years. For OHIP-9, the 50–60 and ≥60-year age groups had higher scores than the 30–45-year age group. In contrast, no age-related significant differences were found for OHIP-8, OHIP-10, OHIP-11, OHIP-12, OHIP-13, or OHIP-14 items (*p* > 0.05). Detailed item-level comparisons, including post hoc pairwise results, are presented in Supplementary Table S3a,b.

### 3.5. Education

Statistically significant differences according to educational level were identified for several periodontal awareness indicators, self-reported symptoms, and oral health behaviors. Higher educational attainment was associated with more frequent reporting of gingival swelling, greater recognition of gingival bleeding as a sign of disease, more regular dental visits, a higher prevalence of previous professional dental scaling, and greater use of adjunctive oral hygiene products. Knowledge of the preventive role of professional dental scaling and of the association between periodontal diseases and systemic diseases also increased with educational level. However, self-reported gingival bleeding during toothbrushing did not differ significantly according to educational level (*p* = 0.212).

Only variables showing statistical significance in at least one comparison or having direct clinical relevance are presented.

With respect to OHIP items, significant differences according to educational level were detected only in the OHIP-2 and OHIP-5 items (*p* < 0.05). OHIP-2 scores were higher in the primary education and doctorate groups, whereas OHIP-5 scores were higher among individuals with bachelor’s degrees or higher. No significant differences were observed in OHIP-3, OHIP-4, OHIP-6, or OHIP-8 to OHIP-14 items (*p* > 0.05). (Detailed item-level comparisons, including post hoc pairwise results, are presented in Supplementary Tables S5 and S6.) Regarding the study-specific clinical periodontal status categories, a significant difference was detected according to educational level; the proportion of primary school graduates was higher in the clinical periodontal health group, whereas the proportions of individuals with bachelor’s degrees or higher were higher in the clinical condition consistent with gingivitis and clinical condition consistent with periodontitis groups (*p* < 0.001).

### 3.6. Relationships Between Periodontal Awareness and Clinical Findings

At baseline, several self-reported periodontal symptoms were significantly associated with clinical periodontal indices. Individuals reporting gingival bleeding had significantly higher gingival index and bleeding index values, whereas individuals reporting gingival swelling had significantly higher gingival index, bleeding index, plaque index, and Ramfjord periodontal index values. Similarly, individuals reporting halitosis had significantly higher gingival index, bleeding index, and plaque index values.

Individuals who did not consider their gums healthy or who were unsure about their periodontal health had higher plaque index and Ramfjord periodontal index values. In addition, all periodontal indices were significantly higher among individuals who had not previously undergone professional dental scaling. Perceived need for periodontal treatment was significantly associated only with the gingival index.

In contrast, several indicators of periodontal awareness and oral health behaviors did not differ significantly across the study-specific clinical periodontal status categories. Regular dental visits, use of adjunctive oral hygiene products, and several knowledge-related variables did not consistently distinguish the clinical periodontal status categories. Detailed comparisons among periodontal awareness, self-reported symptoms, oral health behaviors, and clinical periodontal indices are presented in the Supplementary Tables S11 and S12.

### 3.7. Oral Health-Related Quality of Life and Clinical Findings

At baseline, associations between OHIP-14 item scores and clinical periodontal parameters were generally weak. A weak positive correlation was found between the OHIP-4 item score and the gingival index (Spearman’s rho = 0.104; *p* = 0.033). The OHIP-5 item score showed a weak positive correlation with the plaque index (rho = 0.106; *p* = 0.029). The OHIP-6 item score showed weak positive correlations with the bleeding index (rho = 0.129; *p* = 0.008) and plaque index (rho = 0.118; *p* = 0.015). No statistically significant associations were identified between the remaining OHIP-14 items and clinical periodontal parameters. The complete correlation analyses are presented in Supplementary Table S7.

For most OHIP-14 items, no statistically significant differences were observed across the study-specific clinical periodontal status categories. Among the OHIP-14 items, only the OHIP-6 item score differed significantly across the clinical periodontal status categories, with the highest mean score observed in the gingivitis-compatible clinical status group (*p* = 0.018; Table 4). Complete item-level comparisons are presented in Supplementary Table S5c.

Overall, associations between oral health-related quality of life and objective clinical periodontal findings were weak and inconsistent.

## 4. Discussion

The principal contribution of this observational study is the integrated evaluation of periodontal awareness, self-reported symptoms, oral health behaviors, objective clinical periodontal findings, and oral health-related quality of life within the same sample of Turkish Cypriot adults. In addition, short-term clinical changes following standardized professional tooth cleaning and oral hygiene instruction were examined within the same study framework. The main findings showed that certain self-reported symptoms, including gingival bleeding, swelling, and halitosis, were associated with clinical indicators of inflammation, whereas periodontal knowledge and preventive oral health behaviors were not always consistently associated with clinical periodontal status. Associations between OHIP-14 items and objective clinical periodontal findings were generally weak, while significant reductions in the main clinical periodontal indices were observed at the three-week follow-up. Thus, the study’s original contribution lies not in evaluating a single relationship, but in examining these patient-reported and clinically measured variables together within the same population.

At the three-week follow-up after the standardized professional tooth cleaning and oral hygiene instruction protocol, significant reductions were observed in the gingival index, plaque index, bleeding index, and Ramfjord periodontal index. These findings are consistent with previous studies reporting reductions in plaque accumulation and gingival inflammation following the combined use of professional mechanical plaque control and oral hygiene instruction [17,18,19,20,21]. The three-week follow-up period was selected to examine early changes in plaque-related clinical inflammatory indicators after standardized professional care rather than to evaluate definitive periodontal healing or the long-term durability of treatment. Therefore, although the observed reductions indicate favorable short-term clinical changes, they should not be interpreted as evidence of the independent effectiveness of the intervention because the study did not include a non-intervention control group.

One of the most noteworthy findings of this study was that, although partial concordance existed between self-reported periodontal symptoms and clinical periodontal findings, periodontal awareness indicators and oral health behaviors were not always consistently associated with clinical periodontal status. Symptoms such as gingival bleeding, gingival swelling, and halitosis were associated with indicators of clinical inflammation; however, regular dental visits, use of adjunctive oral hygiene products, and level of knowledge about periodontal diseases often did not significantly differentiate clinical periodontal status categories. This suggests that important differences may exist between the presence of periodontal health behaviors and the effectiveness of those behaviors. In addition, the fact that current clinical status reflects not only current behaviors but also past oral hygiene habits, previous periodontal treatment history, biological susceptibility, and individual risk factors may explain this discrepancy [22,23,24].

The higher prevalence of previous professional dental scaling in the gingivitis-compatible and periodontitis-compatible clinical status groups should not be interpreted as indicating that professional cleaning causes an unfavorable periodontal condition. This finding may be explained by the greater likelihood that individuals with previous periodontal symptoms or clinical problems sought professional care. However, because the timing and indication for previous dental scaling and subsequent personal oral care behaviors were not assessed, the cross-sectional data do not permit determination of the direction or causality of this association. These findings are consistent with previous studies showing that individuals’ self-assessments of their periodontal health do not always correspond with their clinically determined periodontal status [7,8]. The incomplete concordance observed in this study supports the combined use of self-reported information and objective clinical evaluations rather than reliance solely on subjective awareness or knowledge measures.

When demographic variables were evaluated, women exhibited a more favorable profile than men in terms of periodontal awareness and preventive oral health behaviors. Regular dental visits, professional dental scaling, and use of adjunctive oral care products were more common among women. These findings are consistent with previous studies reporting that women have higher oral health literacy and stronger preventive health behaviors [25,26]. In contrast, the more frequent reporting of gingival bleeding during toothbrushing among men suggests that clinical signs of periodontal inflammation may be more common in this group [27]. However, the fact that higher levels of awareness among women were not always associated with better clinical periodontal status once again indicates that periodontal health cannot be explained solely by behavioral indicators.

Age and educational level were also found to influence periodontal awareness. As educational level increased, the rates of recognizing signs of periodontal disease, knowing the importance of professional care, and using adjunctive oral care products increased [28,29]. Similarly, awareness of the relationship between periodontal diseases and systemic diseases was higher among individuals with higher educational levels [30]. Nevertheless, it is noteworthy that higher levels of knowledge and more favorable oral health behaviors did not always parallel clinical periodontal status. This indicates that knowledge acquisition alone may not result in behavioral change or biological improvement, and that periodontal health is shaped by the interaction of numerous behavioral, environmental, and biological factors [8]. The findings concerning educational level and symptom reporting should be interpreted cautiously. Although self-reported gingival bleeding during toothbrushing did not differ significantly according to educational level, reporting of gingival swelling and recognition of gingival bleeding as a sign of disease differed across educational categories. Therefore, higher educational attainment should not be interpreted as a factor that biologically increases the occurrence of overt periodontal symptoms. Rather, education may influence the recognition, interpretation, and reporting of symptoms. The observed associations may also have been affected by clinical periodontal status, previous use of dental services, and unequal sizes of the educational groups. Because these comparisons were not adjusted for confounding factors, no independent or causal effect of educational level on symptom reporting can be inferred.

Another important finding of this study was the generally weak association between oral health-related quality of life and clinical periodontal findings. Only a limited number of weak correlations were identified between OHIP-14 items and clinical periodontal parameters, and only the OHIP-6 item score differed significantly across the study-specific clinical periodontal status categories. These findings may partly reflect the fact that OHIP-14 is a generic oral health-related quality-of-life measure that evaluates not only periodontal symptoms but also the functional, physical, psychological, and social effects of oral health within a broad framework. In addition, the relatively small number of participants in the periodontitis-compatible group and the predominance of participants with clinical periodontal health or gingivitis-compatible status may have reduced the strength of the observed associations. Particularly among individuals with early or moderate clinical findings, periodontal changes may not yet have been perceived as a marked impairment in daily life [9,31,32].

Because OHIP-14 is not specific to periodontal disease and assesses general oral health effects, it may have had reduced sensitivity to the more limited effects of early or mild periodontal changes on quality of life, which may partly explain the weak associations observed with clinical parameters. It should also be noted that OHIP-14 was administered only at baseline because treatment-related change in OHRQoL was not defined as a follow-up outcome in the prespecified study protocol. Different reference periods and reassessment intervals have been used in OHIP studies, and no single interval is appropriate for all study designs [33]. In the present study, reassessment after three weeks was excluded because of the potential temporal overlap between experiences reported at baseline and follow-up. Therefore, the findings reflect only the cross-sectional relationships between baseline OHRQoL and the other study variables.

Although the literature reports that oral health-related quality of life is adversely affected as periodontitis severity increases, it has also been shown that this relationship does not emerge in the same manner in every individual [11,34,35,36,37]. Among individuals with clinically similar periodontal findings, important differences may exist in symptom perception, psychological resilience, esthetic expectations, and health awareness. These interindividual differences may partly explain why associations between OHIP-14 items and clinical periodontal findings were limited and generally weak in the present study.

Overall, this study demonstrates that periodontal health consists not only of clinical indicators or individual levels of awareness. Although periodontal awareness, oral health behaviors, clinical periodontal status, and oral health-related quality of life are interrelated, they did not demonstrate consistent concordance across all comparisons. The incomplete concordance between self-reported awareness and objective clinical status indicates that relying solely on self-report measures may be insufficient for the assessment of periodontal diseases. Therefore, periodontal health programs should address not only the enhancement of individual awareness, but also regular professional follow-up, approaches that support behavioral change, and objective clinical evaluations.

### 4.1. Clinical Implications

The findings of this study indicate that relying solely on individual awareness levels or self-reported symptoms may be insufficient in periodontal health assessments. Although some symptoms such as gingival bleeding, swelling, and halitosis were associated with clinical inflammation, the level of knowledge about periodontal diseases and preventive oral health behaviors did not always parallel clinical periodontal status. Therefore, regular professional periodontal assessments are of major importance for the early recognition and effective management of periodontal diseases.

Furthermore, the weak relationship between oral health-related quality of life and clinical periodontal status suggests that individuals may not always accurately perceive the effects of periodontal disease. This indicates that, especially in preventive dentistry, patient education should focus not only on the transfer of information but also on approaches that support behavioral change. These findings support integrating patient education that promotes behavioral change with regular clinical follow-up in future community-based periodontal health programs.

### 4.2. Limitations

This study has several limitations. First, in accordance with the prespecified scope of the study, OHIP-14 was administered only at baseline because potential changes in OHRQoL after the standardized professional care protocol were not planned as a follow-up outcome. Reassessment after three weeks was excluded because the short interval could create temporal overlap between experiences reported at baseline and follow-up. Nevertheless, the absence of repeated OHRQoL assessment prevented evaluation of potential changes in perceived oral health effects after professional dental scaling. Furthermore, because OHIP-14 is a generic rather than periodontal disease-specific OHRQoL instrument, its sensitivity for detecting the limited effects associated with early or mild periodontal changes may have been reduced.

Second, the follow-up period was limited to three weeks. Although this period allowed assessment of the short-term inflammatory response, it does not permit conclusions regarding the long-term clinical outcomes and sustainability of periodontal treatment. In addition, the study did not include a non-intervention control group. Therefore, the observed pre–post reductions do not establish the independent effectiveness of the standardized professional care protocol.

Another limitation of the study is that full-mouth clinical attachment level measurements and standardized radiographic bone-loss assessments were not included in the study protocol. Therefore, participants were evaluated using study-specific clinical periodontal status categories rather than definitive periodontal diagnoses according to the 2017 periodontal classification. The findings should be interpreted within this methodological framework.

In addition, because the study population consisted of individuals who agreed to attend a dental clinic, the possibility of selection bias cannot be completely excluded. Moreover, the relatively small number of participants aged 60 years and older and the relatively small size of the clinical condition consistent with periodontitis group may have limited statistical power in some subgroup analyses. Because the study included only adults aged 30 years and older, the findings cannot be generalized to individuals younger than 30 years.

Future studies including longer follow-up periods, comprehensive periodontal diagnostic assessments, and repeated quality-of-life measurements will contribute to a more comprehensive understanding of the relationships among periodontal awareness, clinical status, and quality of life.

## 5. Conclusions

The findings of this observational study indicate that periodontal awareness, oral health behaviors, clinical periodontal findings, and oral health-related quality of life did not demonstrate consistent concordance across all comparisons among Turkish Cypriot adults. Although subjective symptoms such as gingival bleeding, gingival swelling, and halitosis were associated with some clinical periodontal parameters, knowledge about periodontal diseases and preventive oral health behaviors were not always consistently associated with clinical periodontal status.

As a secondary and descriptive finding, significant reductions in clinical periodontal parameters were observed three weeks after the standardized professional tooth cleaning and oral hygiene instruction protocol. However, because the study did not include a non-intervention control group, these findings should be interpreted as favorable short-term pre–post changes rather than evidence of the independent effectiveness of the professional care protocol. However, associations between oral health-related quality of life and objective periodontal status were generally weak, indicating that individuals may not always perceive their clinical periodontal status as a marked deterioration in quality of life.

One important finding of the study was the incomplete concordance between self-reported periodontal awareness and objective clinical periodontal status. This finding indicates that self-reported symptoms or awareness measures alone may be insufficient for periodontal assessment and supports the combined use of regular professional follow-up and objective clinical evaluations.

In conclusion, strategies aimed at improving periodontal health should focus not only on increasing knowledge levels, but also on holistic approaches that support behavioral change, ensure continuity of regular professional care, and incorporate clinical evaluations.

## Figures and Tables

**Table 1:** Demographic, behavioral, and clinical characteristics of the study population.

Variable	*n* (%)
Total participants	423 (100.0)
Sex—Male	134 (31.7)
Sex—Female	289 (68.3)
Age 30–45 years	274 (64.8)
Age 46–50 years	66 (15.6)
Age 51–60 years	60 (14.2)
Age > 60 years	23 (5.4)
Education—Primary school	24 (5.7)
Education—High school	102 (24.1)
Education—Bachelor’s degree	206 (48.7)
Education—Master’s degree	71 (16.8)
Education—Doctorate	20 (4.7)
Regular dental visits, yes	185 (43.8)
Previous professional dental scaling, yes	317 (74.9)
Use of dental floss/interdental brush, yes	151 (35.7)
Clinical periodontal health	204 (48.2)
Clinical condition consistent with gingivitis	179 (42.3)
Clinical condition consistent with periodontitis	40 (9.5)

**Table 2:** Changes in the main clinical periodontal parameters from baseline to the three-week follow-up after the standardized professional tooth cleaning and oral hygiene instruction protocol.

Clinical Parameter	T0, Mean ± SD; Median (Min–Max)	T3, Mean ± SD; Median (Min–Max)	*p*-Value
Gingival index	1.02 ± 1.01; 1 (0–3)	0.44 ± 0.64; 0 (0–2)	<0.001
Bleeding on probing, %	6.20 ± 6.64; 4 (0–23)	5.30 ± 5.62; 4 (0–19)	<0.001
Plaque index	1.30 ± 1.08; 1 (0–3)	0.60 ± 0.77; 0 (0–2)	<0.001
Ramfjord periodontal index	1.50 ± 1.68; 1 (0–6)	0.91 ± 1.32; 0 (0–5)	<0.001

Data are presented as mean ± standard deviation and median (minimum–maximum). T0 and T3 measurements were compared using the Wilcoxon signed-rank test. Paired analyses were performed for the 423 participants with measurements available at both T0 and T3. Detailed site-specific probing depth results are presented in Supplementary Table S1.

**Table 3:** Summary of findings on periodontal awareness, self-reported symptoms, and oral health behaviors according to sex, age group, and educational level.

Variable	Sex *p*-Value	Age *p*-Value	Education *p*-Value
Gingival bleeding	0.077	0.248	0.018
Gingival inflammation/infection	0.401	0.036	0.188
Gingival pain	0.046	0.919	0.092
Gingival swelling	0.462	0.344	<0.001
Gingival bleeding during toothbrushing	0.015	0.041	0.212
Awareness that gingival bleeding may indicate disease	0.063	0.339	0.002
Regular dental visits	0.008	0.350	<0.001
Previous professional dental scaling	0.040	0.674	<0.001
Use of dental floss/interdental brush	0.012	0.352	0.023
Awareness that professional dental scaling may help prevent periodontal diseases	0.039	0.625	0.009
Awareness of the association between periodontal diseases and cardiovascular disease/diabetes mellitus	0.035	0.204	<0.001
Smoking status	0.810	0.004	0.793

*p*-values were calculated using Fisher’s exact test. Statistically significant values are shown in bold. Only variables that showed statistical significance in at least one comparison are presented. Detailed group distributions are provided in Supplementary Tables S2–S4.

**Table 4:** Variables showing statistically significant differences across the study-specific clinical periodontal status categories.

Variable	Clinical Periodontal Health (*n* = 204)	Gingivitis-Compatible Clinical Status (*n* = 179)	Periodontitis-Compatible Clinical Status (*n* = 40)	*p*-Value
Education, *n* (%)				<0.001
Primary school	19 (9.3)	5 (2.8)	0 (0.0)	
High school	59 (28.9)	39 (21.8)	4 (10.0)	
Bachelor’s degree	79 (38.7)	102 (57.0)	25 (62.5)	
Master’s degree	37 (18.1)	26 (14.5)	8 (20.0)	
Doctorate	10 (4.9)	7 (3.9)	3 (7.5)	
Gingival swelling—yes, *n* (%)	76 (37.3)	89 (49.7)	24 (60.0)	0.006
Previous professional dental scaling—yes, *n* (%)	139 (68.1)	143 (80.3)	35 (87.5)	0.004
OHIP-6 item score, mean ± SD; median (min–max)	2.12 ± 1.01; 2 (1–5)	2.40 ± 0.96; 2 (1–5)	2.20 ± 1.22; 2 (1–5)	0.018

Categorical variables were compared using Fisher’s exact test, and the OHIP-6 item score was compared using the Kruskal–Wallis test. Data are presented as *n* (%) or mean ± standard deviation and median (minimum–maximum), as appropriate. Only variables showing statistically significant differences are presented in the main table. All comparisons are provided in Supplementary Tables S8–S10.

## Data Availability

The data are not publicly available due to ethical and privacy restrictions but may be made available from the corresponding author upon reasonable request.

## Supplementary Materials

The following supporting information can be downloaded at: https://www.mdpi.com/article/10.3390/healthcare14162591/s1, Table S1: Changes in site-specific probing pocket depth measurements in Ramfjord teeth and overall periodontal clinical indices from T0 to T3; Table S2: Full distribution of periodontal awareness indicators and oral-health behaviors according to sex; Table S3: Full distribution of periodontal awareness indicators and oral-health behaviors according to age group; Table S4: Full distribution of periodontal awareness indicators and oral-health behaviors according to educational level; Table S5: Full item-level comparisons of OHIP-14 scores according to sex, age group, and educational level: OHIP-1 to OHIP-7; Table S6: Full item-level comparisons of OHIP-14 scores according to sex, age group, and educational level: OHIP-8 to OHIP-14; Table S7: Spearman correlation analysis between OHIP-14 item scores and periodontal clinical parameters at baseline; Table S8: Full comparisons across study-specific clinical periodontal status categories: demographic variables; Table S9: Full comparisons across study-specific clinical periodontal status categories: awareness and behavior variables; Table S10: Full comparisons across study-specific clinical periodontal status categories: OHIP-14 item scores; Table S11: Full comparisons between periodontal awareness/self-reported symptoms/oral-health behaviors and clinical periodontal indices at T0; Table S12: Full comparisons between periodontal awareness/self-reported symptoms/oral-health behaviors and clinical periodontal indices at T3.

## Abbreviations

The following abbreviations are used in this manuscript:

WHO	World Health Organization
UNC-15	University of North Carolina–15 periodontal probe
GI	Gingival Index
PI	Plaque Index
BOP	Bleeding on Probing
RPI	Ramfjord Periodontal Index
PPD	Periodontal Pocket Depth
T0	Baseline measurement
T3	3-week follow-up
OHIP-14	Oral Health Impact Profile-14
OHRQoL	Oral Health-Related Quality of Life
