# Supplementary material for: Relationships Among Periodontal Awareness, Clinical Findings, and Oral Health-Related Quality of Life in Turkish Cypriot Adults
Source: Healthcare (Basel). 2026 Aug 18;14(16):2591. doi: 10.3390/healthcare14162591 (PMC13512849; doi:10.3390/healthcare14162591)
Supplement: Supplementary file 1 [file healthcare-14-02591-s001.zip › healthcare-4389633-supplementary.pdf]

Supplementary Table S1. Changes in site-specific probing pocket depth measurements in Ramfjord teeth and overall periodontal clinical indices from T0 to T3.

**Mean ± SD**<br>**Med. (Min.-Max.)**	**T0**	**T3**	**p**
**16 DISTOBUCCAL (mm)**	2.87 ± 1.15<br>3- (1-12)	2.76 ± 0.91<br>3- (1-10)	**<0.001**
**16 MID BUCCAL (mm)**	1.73 ± 0.83<br>2- (0-10)	1.72 ± 0.76<br>2- (0-8)	0.157
**16 MESIOBUCCAL (mm)**	2.60 ± 0.88<br>2- (1-8)	2.57 ± 0.81<br>2- (1-6)	**0.005**
**16 DISTOPALATAL (mm)**	2.94 ± 1.21<br>3- (1-14)	2.82 ± 0.96<br>3- (1-12)	**<0.001**
**16 MID PALATAL (mm)**	1.9 ± 0.83<br>2- (0-11)	1.89 ± 0.76<br>2- (0-9)	0.157
**16 MESIOPALATAL (mm)**	2.8 ± 1.02<br>3- (0-7)	2.71 ± 0.80<br>3- (0-5)	**<0.001**
**21DISTOLABIAL (mm)**	1.97 ± 0.91<br>2- (0-6)	1.94 ± 0.83<br>2- (0-4)	**0.015**
**21 MID LABIAL (mm)**	1.56 ± 0.88<br>2- (0-7)	1.55 ± 0.82<br>2- (0-5)	0.102
**21 MESIOLABIAL (mm)**	2.02 ± 0.98<br>2- (0-8)	1.99 ± 0.89<br>2- (0-6)	**0.009**
**21 DISTOPALATAL (mm)**	2.15 ± 0.98<br>2- (0-9)	2.12 ± 0.85<br>2- (0-7)	**0.004**
**21 MID PALATAL (mm)**	1.66 ± 0.88<br>2- (0-8)	1.65 ± 0.82<br>2- (0-6)	0.102
**21 MESIOPALATAL (mm)**	2.1 ± 1.04<br>2- (0-10)	2.06 ± 0.89<br>2- (0-8)	**0.001**
**24 DISTOBUCCAL (mm)**	2.34 ± 0.76<br>2- (0-6)	2.33 ± 0.72<br>2- (0-4)	0.102
**24 MID BUCCAL (mm)**	1.73 ± 0.79<br>2- (0-7)	1.72 ± 0.75<br>2- (0-5)	0.157
**24 MESIOBUCCAL (mm)**	2.48 ± 0.99<br>2- (0-9)	2.43 ± 0.83<br>2- (0-7)	**<0.001**
**24 DISTOPALATAL (mm)**	2.44 ± 0.90<br>2- (0-7)	2.40 ± 0.80<br>2- (0-5)	**0.001**
**24 MID PALATAL (mm)**	1.82 ± 0.81<br>2- (0-8)	1.82 ± 0.77<br>2- (0-6)	0.180
**24 MESIOPALATAL (mm)**	2.39 ± 1<br>2- (0-9)	2.35 ± 0.89<br>2- (0-7)	**0.002**
**36 DISTOBUCCAL (mm)**	2.46 ± 0.87<br>2- (1-7)	2.42 ± 0.76<br>2- (1-5)	**0.003**

**36 MID BUCCAL (mm)**	1.84 ± 0.83<br>2- (0-7)	1.82 ± 0.75<br>2- (0-5)	**0.038**
**36MESIOBUCCAL (mm)**	2.58 ± 1.04<br>2- (1-9)	2.52 ± 0.85<br>2- (1-7)	**<0.001**
**36 DISTOLINGUAL (mm)**	2.59 ± 0.94<br>2- (1-8)	2.53 ± 0.78<br>2- (1-6)	**<0.001**
**36 MID LINGUAL (mm)**	1.95 ± 0.84<br>2- (0-8)	1.94 ± 0.77<br>2- (0-6)	0.083
**36 MESIOLINGUAL (mm)**	2.62 ± 1.04<br>3- (0-7)	2.55 ± 0.86<br>3- (0-5)	**<0.001**
**41 DISTOLABIAL (mm)**	1.99 ± 0.75<br>2- (0-6)	1.98 ± 0.73<br>2- (0-4)	0.180
**41 MID LABIAL (mm)**	1.44 ± 0.8<br>1- (0-6)	1.43 ± 0.75<br>1- (0-4)	0.157
**41 MESIOLABIAL (mm)**	1.93 ± 0.78<br>2- (0-6)	1.92 ± 0.75<br>2- (0-4)	0.180
**41 DISTOLINGUAL (mm)**	1.95 ± 0.8<br>2- (0-7)	1.94 ± 0.75<br>2- (0-5)	0.157
**41 MID LINGUAL (mm)**	1.57 ± 0.77<br>2- (0-6)	1.57 ± 0.72<br>2- (0-4)	0.157
**41 MESIOLINGUAL (mm)**	1.92 ± 0.82<br>2- (0-8)	1.91 ± 0.77<br>2- (0-6)	0.157
**44 DISTOBUCCAL (mm)**	2.26 ± 0.9<br>2- (0-7)	2.23 ± 0.83<br>2- (0-5)	**0.008**
**44 MID BUCCAL (mm)**	1.54 ± 0.74<br>2- (0-5)	1.54 ± 0.73<br>2- (0-4)	0.317
**44 MESIOBUCCAL (mm)**	2.25 ± 0.83<br>2- (0-7)	2.23 ± 0.77<br>2- (0-5)	**0.038**
**44 DISTOLINGUAL (mm)**	2.40 ± 0.98<br>2- (0-7)	2.34 ± 0.83<br>2- (0-5)	**<0.001**
**44 MID LINGUAL (mm)**	1.76 ± 0.71<br>2- (0-5)	1.76 ± 0.7<br>2- (0-4)	0.317
**44 MESIOLINGUAL (mm)**	2.38 ± 0.93<br>2- (0-9)	2.34 ± 0.82<br>2- (0-7)	**0.002**
**GINGIVAL INDEX**	1.02 ± 1.01<br>1- (0-3)	0.44 ± 0.64<br>0- (0-2)	**<0.001**
**BLEEDING ON PROBING %**	6.20 ± 6.64<br>4- (0-23)	5.30 ± 5.62<br>4- (0-19)	**<0.001**
**PLAQUE INDEX**	1.30 ± 1.08<br>1- (0-3)	0.60 ± 0.77<br>0- (0-2)	**<0.001**
**RAMFJORD PERIODONTAL INDEX**	1.50 ± 1.68<br>1- (0-6)	0.91 ± 1.32<br>0- (0-5)	**<0.001**

Supplementary Table S2. Full distribution of periodontal awareness indicators and oral-health behaviors according to sex.

Sex		Male		Female		
		N	%	N	%	p
Gingival bleeding	Yes	90	67.2%	219	75.8%	0.077
	No	44	32.8%	70	24.2%	
Gingival inflammation/infection	Yes	62	46.3%	121	41.9%	0.401
	No	72	53.7%	168	58.1%	
Gingival pain	Yes	61	45.5%	163	56.4%	**0.046**
	No	73	54.5%	126	43.6%	
Gingival swelling	Yes	56	41.8%	133	46.0%	0.462
	No	78	58.2%	156	54.0%	
Gingival itching	Yes	11	8.2%	22	7.6%	0.847
	No	123	91.8%	267	92.4%	
Cold/hot tooth sensitivity	Yes	41	30.6%	118	40.8%	0.052
	No	93	69.4%	171	59.2%	
Perceived gingival health	Yes	66	49.6%	133	46.2%	0.493
	No	24	18.0%	45	15.6%	
	Not Sure	43	32.3%	110	38.2%	
Toothbrushing frequency	Once a day	42	31.3%	62	21.5%	-
	Twice a day	69	51.5%	203	70.5%	
	3 times a day or more	6	4.5%	17	5.9%	
	Sometimes	15	11.2%	6	2.1%	
	Never	2	1.5%	0	0.0%	
Awareness that gingival bleeding may indicate disease	Yes	81	60.4%	206	71.8%	0.063
	No	29	21.6%	42	14.6%	
	Don't know	24	17.9%	39	13.6%	
Halitosis	Yes	23	17.2%	35	12.2%	0.353
	No	60	44.8%	138	48.1%	
	Sometimes	51	38.1%	114	39.7%	
Gingival bleeding during toothbrushing	Yes	38	28.4%	61	21.3%	**0.015**
	No	41	30.6%	69	24.0%	
	Sometimes	55	41.0%	157	54.7%	
Regular dental visits	Yes	46	34.3%	139	48.3%	**0.008**
	No	88	65.7%	149	51.7%	
Previous professional dental scaling	Yes	92	68.7%	225	78.1%	**0.040**
	No	42	31.3%	63	21.9%	

Smoking status	5 cigarettes/day	6	4.5%	22	7.6%	0.810
	10 cigarettes/day	10	7.5%	32	11.1%	
	≥1 pack/day	40	29.9%	31	10.8%	
	Non-smoker	78	58.2%	203	70.5%	
Use of dental floss or interdental brushes	Yes	36	27.1%	115	39.9%	**0.012**
	No	97	72.9%	173	60.1%	
Awareness that professional dental scaling may help prevent periodontal diseases	Yes	57	42.5%	170	59.0%	**0.039**
	No	40	29.9%	44	15.3%	
	Don’t know	37	27.6%	74	25.7%	
Awareness of the association between periodontal diseases and cardiovascular disease/diabetes mellitus	Yes	53	40.2%	153	53.3%	**0.035**
	No	51	38.6%	84	29.3%	
	Not sure	28	21.2%	50	17.4%	
Perceived need for periodontal treatment	Yes	112	83.6%	259	90.6%	0.054
	No	5	3.7%	5	1.7%	
	Not sure	17	12.7%	22	7.7%	

Percentages were calculated using the number of valid responses for each variable; denominators may vary because of item-level missing responses.
The first six variables represent responses to the multiple-response question asking what first comes to mind when participants hear the term “gingival disease”; they do not represent current self-reported symptoms.
The p-value for toothbrushing frequency was not calculated because of sparse cell counts.

Supplementary Table S3. Full distribution of periodontal awareness indicators and oral-health behaviors according to age group.

Age		30–45		46–50		51–60		>60		
		**N**	**%**	**N**	**%**	**N**	**%**	**N**	**%**	**p**
Gingival bleeding	Yes	192	70.10%	55	83.30%	45	75.00 %	17	73.90%	0.248
	No	82	29.90%	11	16.70%	15	25.00 %	6	26.10%	
Gingival inflammation/infection	Yes	114	41.60%	25	37.90%	27	45.00 %	17	73.90%	**0.036**
	No	160	58.40%	41	62.10%	33	55.00 %	6	26.10%	
Gingival pain	Yes	143	52.20%	37	56.10%	33	55.00 %	11	47.80%	0.919
	No	131	47.80%	29	43.90%	27	45.00 %	12	52.20%	
Gingival swelling	Yes	120	43.80%	38	57.60%	26	43.30 %	5	21.70%	0.344
	No	154	56.20%	28	42.40%	34	56.70 %	18	78.30%	
Gingival itching	Yes	24	8.80%	4	6.10%	3	5.00 %	2	8.70%	0.444
	No	250	91.20%	62	93.90%	57	95.00 %	21	91.30%	
Cold/hot tooth sensitivity	Yes	104	38.00%	24	36.40%	26	43.30 %	5	21.70%	0.598
	No	170	62.00%	42	63.60%	34	56.70 %	18	78.30%	
Perceived gingival health	Yes	126	46.30%	33	50.00%	29	48.30 %	11	47.80%	0.518
	No	43	15.80%	11	16.70%	10	16.70 %	5	21.70%	
	Not sure	103	37.90%	22	33.30%	21	35.00 %	7	30.40%	
Toothbrushing frequency	Once a day	64	23.40%	12	18.20%	21	35.00 %	7	30.40%	-
	Twice a day	180	65.90%	47	71.20%	33	55.00 %	12	52.20%	

	3 times a day or more	15	5.50%	3	4.50%	4	6.70 %	1	4.30%	
	Sometimes	12	4.40%	4	6.10%	2	3.30 %	3	13.00%	
	Never	2	0.70%	0	0.00%	0	0.00 %	0	0.00%	
Awareness that gingival bleeding may indicate disease	Yes	178	65.20%	49	74.20%	44	74.60 %	16	69.60%	0.339
	No	52	19.00%	9	13.60%	8	13.60 %	2	8.70%	
	Don't know	43	15.80%	8	12.10%	7	11.90 %	5	21.70%	
Halitosis	Yes	42	15.40%	7	10.60%	7	11.70 %	2	8.70%	0.688
	No	124	45.60%	31	47.00%	29	48.30 %	14	60.90%	
	Sometimes	106	39.00%	28	42.40%	24	40.00 %	7	30.40%	
Gingival bleeding during toothbrushing	Yes	73	26.80%	13	19.70%	9	15.00 %	4	17.40%	**0.041**
	No	72	26.50%	15	22.70%	15	25.00 %	8	34.80%	
	Sometimes	127	46.70%	38	57.60%	36	60.00 %	11	47.80%	
Regular dental visits	Yes	121	44.30%	31	47.00%	27	45.00 %	6	26.10%	0.350
	No	152	55.70%	35	53.00%	33	55.00 %	17	73.90%	
Previous professional dental scaling	Yes	202	74.00%	49	74.20%	52	86.70 %	14	60.90%	0.674
	No	71	26.00%	17	25.80%	8	13.30 %	9	39.10%	
Smoking status	5 cigarettes/day	21	7.70%	4	6.10%	1	1.70 %	2	8.70%	**0.004**
	10 cigarettes/day	32	11.70%	8	12.10%	1	1.70 %	1	4.30%	
	≥1 pack/day	53	19.40%	8	12.10%	6	10.00 %	4	17.40%	

	Non-smoker	167	61.20%	46	69.70%	52	86.70 %	16	69.60%	
Use of dental floss or interdental brushes	Yes	95	34.90%	31	47.00%	23	38.30 %	2	8.70%	0.352
	No	177	65.10%	35	53.00%	37	61.70 %	21	91.30%	
Awareness that professional dental scaling may help prevent periodontal diseases	Yes	148	54.20%	36	54.50%	33	55.00 %	10	43.50%	0.625
	No	48	17.60%	11	16.70%	17	28.30 %	8	34.80%	
	Don't know	77	28.20%	19	28.80%	10	16.70 %	5	21.70%	
Awareness of the association between periodontal diseases and cardiovascular disease/diabetes mellitus	Yes	129	47.40%	33	50.00%	32	55.20 %	12	52.20%	0.204
	No	88	32.40%	22	33.30%	17	29.30 %	8	34.80%	
	Not sure	55	20.20%	11	16.70%	9	15.50 %	3	13.00%	
Perceived need for periodontal treatment	Yes	235	86.40%	59	89.40%	55	93.20 %	22	95.70%	0.074
	No	8	2.90%	1	1.50%	1	1.70 %	0	0.00%	
	Not sure	29	10.70%	6	9.10%	3	5.10 %	1	4.30%	

Percentages were calculated using the number of valid responses for each variable; denominators may vary because of item-level missing responses.
The first six variables represent responses to the multiple-response question asking what first comes to mind when participants hear the term "gingival disease"; they do not represent current self-reported symptoms.
The p-value for toothbrushing frequency was not calculated because of sparse cell counts.

Supplementary Table S4. Full distribution of periodontal awareness indicators and oral-health behaviors according to educational level.

Education		Primary School		High School		Undergraduate		Master's		PhD		
		**N**	**%**	**N**	**%**	**N**	**%**	**N**	**%**	**N**	**%**	**p**
Gingival bleeding	Yes	15	62.5%	67	65.7%	155	75.2%	56	78.9%	16	80.0%	**0.018**
	No	9	37.5%	35	34.3%	51	24.8%	15	21.1%	4	20.0%	
Gingival Inflammation/infection	Yes	11	45.8%	49	48.0%	87	42.2%	30	42.3%	6	30.0%	0.188
	No	13	54.2%	53	52.0%	119	57.8%	41	57.7%	14	70.0%	
Gingival pain	Yes	10	41.7%	50	49.0%	112	54.4%	39	54.9%	13	65.0%	0.092
	No	14	58.3%	52	51.0%	94	45.6%	32	45.1%	7	35.0%	
Gingival swelling	Yes	4	16.7%	30	29.4%	108	52.4%	35	49.3%	12	60.0%	**<0.001**
	No	20	83.3%	72	70.6%	98	47.6%	36	50.7%	8	40.0%	
Gingival itching	Yes	1	4.2%	11	10.8%	13	6.3%	8	11.3%	0	0.0%	0.695
	No	23	95.8%	91	89.2%	193	93.7%	63	88.7%	20	100.0%	
Cold/hot tooth sensitivity	Yes	8	33.3%	41	40.2%	83	40.3%	20	28.2%	7	35.0%	0.355
	No	16	66.7%	61	59.8%	123	59.7%	51	71.8%	13	65.0%	
Perceived gingival health	Yes	9	37.5%	39	39.0%	98	47.6%	43	60.6%	10	50.0%	0.050
	No	7	29.2%	22	22.0%	26	12.6%	12	16.9%	2	10.0%	
	Not sure	8	33.3%	39	39.0%	82	39.8%	16	22.5%	8	40.0%	
Toothbrushing frequency	Once a day	6	25.0%	32	31.7%	47	22.8%	15	21.1%	4	20.0%	0.882
	Twice a day	13	54.2%	59	58.4%	141	68.4%	45	63.4%	14	70.0%	

	3 times a day or more	1	4.2%	3	3.0%	11	5.3%	7	9.9%	1	5.0%	
	Sometimes	4	16.7%	5	5.0%	7	3.4%	4	5.6%	1	5.0%	
	Never	0	0.0%	2	2.0%	0	0.0%	0	0.0%	0	0.0%	
Awareness that gingival bleeding may indicate disease	Yes	14	58.3%	58	57.4%	144	70.2%	56	78.9%	15	75.0%	**0.002**
	No	6	25.0%	19	18.8%	32	15.6%	12	16.9%	2	10.0%	
	Don't know	4	16.7%	24	23.8%	29	14.1%	3	4.2%	3	15.0%	
Halitosis	Yes	6	25.0%	15	15.0%	22	10.7%	13	18.3%	2	10.0%	0.368
	No	13	54.2%	49	49.0%	89	43.2%	38	53.5%	9	45.0%	
	Sometimes	5	20.8%	36	36.0%	95	46.1%	20	28.2%	9	45.0%	
Gingival bleeding during toothbrushing	Yes	9	37.5%	27	27.0%	43	20.9%	18	25.4%	2	10.0%	0.212
	No	7	29.2%	22	22.0%	55	26.7%	16	22.5%	10	50.0%	
	Sometimes	8	33.3%	51	51.0%	108	52.4%	37	52.1%	8	40.0%	
Regular dental visits	Yes	7	29.2%	32	31.7%	89	43.2%	45	63.4%	12	60.0%	**<0.001**
	No	17	70.8%	69	68.3%	117	56.8%	26	36.6%	8	40.0%	
Previous professional dental scaling	Yes	12	50.0%	67	66.3%	162	78.6%	61	85.9%	15	75.0%	**<0.001**
	No	12	50.0%	34	33.7%	44	21.4%	10	14.1%	5	25.0%	
Smoking status	5 pieces a day	1	4.2%	3	3.0%	15	7.3%	9	12.7%	0	0.0%	0.793
	10 pieces a day	2	8.3%	14	13.9%	18	8.7%	7	9.9%	1	5.0%	
	1 pack a day or more	6	25.0%	24	23.8%	29	14.1%	8	11.3%	4	20.0%	
	No. I don't use	15	62.5%	60	59.4%	144	69.9%	47	66.2%	15	75.0%	

Use of dental floss or interdental brushes	Yes	5	20.8%	31	31.0%	75	36.4%	32	45.1%	8	40.0%	**0.023**
	No	19	79.2%	69	69.0%	131	63.6%	39	54.9%	12	60.0%	
Awareness that professional dental scaling may help prevent periodontal diseases	Yes	9	37.5%	45	44.6%	112	54.4%	46	64.8%	15	75.0%	**0.009**
	No	10	41.7%	25	24.8%	39	18.9%	8	11.3%	2	10.0%	
	Don’t know	5	20.8%	31	30.7%	55	26.7%	17	23.9%	3	15.0%	
Awareness of the association between periodontal diseases and cardiovascular disease/ diabetes mellitus	Yes	5	20.8%	39	39.0%	102	49.8%	47	67.1%	13	65.0%	**<0.001**
	No	10	41.7%	41	41.0%	62	30.2%	17	24.3%	5	25.0%	
	Not sure	9	37.5%	20	20.0%	41	20.0%	6	8.6%	2	10.0%	
Perceived need for periodontal treatment	Yes	19	79.2%	85	85.0%	187	90.8%	64	91.4%	16	80.0%	0.411
	No	5	20.8%	0	0.0%	2	1.0%	1	1.4%	2	10.0%	
	Not sure	0	0.0%	15	15.0%	17	8.3%	5	7.1%	2	10.0%	

Percentages were calculated using the number of valid responses for each variable; denominators may vary because of item-level missing responses.

The first six variables represent responses to the multiple-response question asking what first comes to mind when participants hear the term “gingival disease”; they do not represent current self-reported symptoms.

Supplementary Table S5. Full item-level comparisons of OHIP-14 scores according to sex, age group, and educational level: OHIP-1 to OHIP-7.

Mean ± SD<br>Med. (Min.-Max.)		OHIP-1	OHIP-2	OHIP-3	OHIP-4	OHIP-5	OHIP-6	OHIP-7
Sex	Male	1.59±0.95<br>1- (1-5)	1.52±0.86<br>1- (1-5)	2.25±0.97<br>2- (1-5)	1.96±0.99<br>2- (1-5)	2.79±1.39<br>3- (1-5)	2.19±1.03<br>2- (1-5)	1.66±0.93<br>1- (1-5)
	Female	1.61±0.95<br>1- (1-5)	1.51±0.82<br>1- (1-5)	2.46±0.96<br>3- (1-5)	2.15±0.97<br>2- (1-5)	3.06±1.23<br>3- (1-5)	2.27±1.02<br>2- (1-5)	1.61±0.88<br>1- (1-5)
	**p[1]**	0.814	0.911	0.052	**0.038**	0.077	0.488	0.520
Age	30 45	1.5±0.85<br>1- (1-5)	1.45±0.77<br>1- (1-5)	2.29±0.98<br>2- (1-5)[a]	2.02±1.01<br>2- (1-5)[a]	2.92±1.27<br>3- (1-5)	2.11±1<br>2- (1-5)[a]	1.61±0.91<br>1- (1-5)
	46–50	1.68±1.01<br>1- (1-5)	1.55±0.86<br>1- (1-5)	2.44±0.86<br>2.5- (1-4)	2.12±0.9<br>2- (1-5)	3.14±1.38<br>3- (1-5)	2.32±1.04<br>2- (1-5)	1.59±0.78<br>1- (1-4)
	51–60	1.82±1.1<br>1- (1-5)	1.63±0.86<br>1- (1-5)	2.85±0.88<br>3- (1-5)[a]	2.35±0.84<br>2- (1-4)[a]	3.15±1.18<br>4- (1-5)	2.6±0.89<br>3- (1-4)[a]	1.62±0.78<br>1- (1-3)
	>60	2.04±1.26<br>1- (1-5)	1.91±1.2<br>1- (1-5)	2.3±1.02<br>3- (1-4)	2.13±1.06<br>2- (1-4)	2.7±1.46<br>3- (1-5)	2.74±1.21<br>3- (1-5)	1.96±1.22<br>1- (1-5)
	**p[2]**	**0.032**	0.089	**<0.001**	**0.035**	0.282	**<0.001**	0.590
Educ-<br>ation	Primary School	1.92±1.14<br>1- (1-5)	1.92±1.06<br>2- (1-5)	2.38±1.06<br>2.5- (1-5)	2.29±1<br>3- (1-4)	2.04±1.27<br>1.5- (1-5)	2.33±1.09<br>3- (1-4)	1.67±0.82<br>1- (1-3)
	High School	1.64±1.08<br>1- (1-5)	1.52±0.89<br>1- (1-5)	2.38±1.03<br>2- (1-5)	2.06±1.12<br>2- (1-5)	2.37±1.33<br>2- (1-5)	2.11±1.03<br>2- (1-5)	1.59±0.96<br>1- (1-5)
	Undergraduate	1.53±0.83<br>1- (1-4)	1.44±0.75<br>1- (1-5)	2.38±0.91<br>2- (1-5)	2.1±0.93<br>2- (1-5)	3.16±1.18<br>4- (1-5)	2.31±1.02<br>2- (1-5)	1.64±0.89<br>1- (1-5)
	Master's	1.59±0.95<br>1- (1-5)	1.48±0.81<br>1- (1-5)	2.38±0.9<br>3- (1-5)	1.96±0.87<br>2- (1-4)	3.49±1.09<br>4- (1-5)	2.21±0.97<br>2- (1-5)	1.63±0.81<br>1- (1-3)
	PhD	1.8±1.11<br>1- (1-4)	1.85±0.93<br>2- (1-4)	2.7±1.3<br>3- (1-5)	2.3±1.03<br>2- (1-5)	3.35±1.14<br>3- (1-5)	2.3±1.08<br>2- (1-5)	1.6±0.99<br>1- (1-4)
	**p[2]**	0.545	**0.032**	0.932	0.433	**<0.001**	0.635	0.811

Mann–Whitney U test was used for comparisons between sex groups, and the Kruskal–Wallis test was used for comparisons among age and educational-level groups. Different superscript letters within the same OHIP-14 item indicate statistically significant pairwise differences identified by Bonferroni-adjusted post-hoc comparisons.

Supplementary Table S6. Full item-level comparisons of OHIP-14 scores according to sex, age group, and educational level: OHIP-8 to OHIP-14.

Mean ± SD<br>Med. (Min.-Max.)		OHIP-8	OHIP-9	OHIP-10	OHIP-11	OHIP-12	OHIP-13	OHIP-14
Sex	Male	1.72±0.89<br>1- (1-5)	2.11±1.05<br>2- (1-5)	1.74±1.03<br>1- (1-5)	1.55±0.85<br>1- (1-4)	1.57±0.79<br>1- (1-4)	1.51±0.85<br>1- (1-5)	1.34±0.66<br>1- (1-4)
	Female	1.84±0.92<br>2- (1-5)	2.18±1<br>2- (1-5)	1.7±0.99<br>1- (1-5)	1.51±0.8<br>1- (1-5)	1.67±0.87<br>1- (1-5)	1.59±0.89<br>1- (1-5)	1.4±0.8<br>1- (1-5)
	**p¹**	0.168	0.404	0.837	0.762	0.352	0.302	0.830
Age	30–45	1.79±0.96<br>1.5- (1-5)	2.06±1.04<br>2- (1-5)	1.72±1.06<br>1- (1-5)	1.56±0.86<br>1- (1-5)	1.61±0.84<br>1- (1-5)	1.53±0.88<br>1- (1-5)	1.37±0.77<br>1- (1-5)
	46–50	1.68±0.73<br>2- (1-3)	2.29±0.86<br>2- (1-5)	1.58±0.86<br>1- (1-4)	1.35±0.62<br>1- (1-4)	1.58±0.8<br>1- (1-4)	1.5±0.79<br>1- (1-4)	1.36±0.62<br>1- (1-3)
	51–60	1.92±0.81<br>2- (1-4)	2.37±0.99<br>2- (1-4)	1.75±0.84<br>2- (1-4)	1.53±0.79<br>1- (1-4)	1.85±0.86<br>2- (1-5)	1.7±0.85<br>2- (1-5)	1.37±0.78<br>1- (1-5)
	>60	1.91±1.04<br>2- (1-4)	2.43±1.16<br>3- (1-4)	1.83±1.11<br>1- (1-4)	1.52±0.85<br>1- (1-4)	1.57±0.99<br>1- (1-4)	1.83±1.07<br>1- (1-4)	1.61±0.99<br>1- (1-4)
	**p²**	0.373	**0.016**	0.494	0.467	0.066	0.121	0.613
Educ-ation	Primary School	2.17±1.01<br>2- (1-4)	2.5±1.1<br>2.5- (1-5)	1.54±0.88<br>1- (1-3)	1.63±0.88<br>1- (1-4)	1.92±0.93<br>2- (1-4)	1.75±0.94<br>1- (1-4)	1.58±0.88<br>1- (1-4)
	High School	1.78±0.95<br>1- (1-5)	2.17±1.07<br>2- (1-5)	1.69±1.05<br>1- (1-5)	1.55±0.84<br>1- (1-5)	1.67±0.92<br>1- (1-5)	1.54±0.89<br>1- (1-5)	1.42±0.83<br>1- (1-5)
	Undergraduate	1.82±0.91<br>2- (1-5)	2.17±0.99<br>2- (1-5)	1.76±1.02<br>1- (1-5)	1.52±0.8<br>1- (1-5)	1.58±0.8<br>1- (1-5)	1.58±0.84<br>1- (1-5)	1.37±0.73<br>1- (1-5)
	Master's	1.66±0.74<br>2- (1-4)	2.06±0.92<br>2- (1-5)	1.61±0.89<br>1- (1-4)	1.38±0.72<br>1- (1-4)	1.61±0.82<br>1- (1-4)	1.42±0.75<br>1- (1-4)	1.27±0.56<br>1- (1-3)
	PhD	1.75±1.12<br>1- (1-5)	1.95±1.23<br>1.5- (1-5)	1.85±1.14<br>1.5- (1-5)	1.75±1.12<br>1- (1-4)	1.85±0.99<br>2- (1-4)	1.85±1.35<br>1- (1-5)	1.45±1.1<br>1- (1-5)
	**p²**	0.255	0.287	0.523	0.427	0.337	0.399	0.529

Mann–Whitney U test was used for comparisons between sex groups, and the Kruskal–Wallis test was used for comparisons among age and educational-level groups. Different superscript letters within the same OHIP-14 item indicate statistically significant pairwise differences identified by Bonferroni-adjusted post-hoc comparisons.

Supplementary Table S7. Spearman correlation analysis between OHIP-14 item scores and periodontal clinical parameters at baseline.

T0		GINGIVAL INDEX	BLEEDING ON PROBING %	PLAQUE INDEX	RAMFJORD PERIODONTAL INDEX
**OHIP-1**	**r**	-0.002	0.031	0.007	0.023
	**p**	0.974	0.526	0.881	0.635
**OHIP-2**	**r**	0.027	0.057	0.085	0.040
	**p**	0.582	0.242	0.083	0.416
**OHIP-3**	**r**	0.053	0.062	0.081	0.070
	**p**	0.279	0.202	0.097	0.151
**OHIP-4**	**r**	**0.104**	0.090	0.088	0.093
	**p**	**0.033**	0.065	0.070	0.056
**OHIP-5**	**r**	0.068	0.075	**0.106**	0.051
	**p**	0.161	0.123	**0.029**	0.295
**OHIP-6**	**r**	0.072	**0.129**	**0.118**	0.071
	**p**	0.137	**0.008**	**0.015**	0.143
**OHIP-7**	**r**	0.040	0.076	0.072	0.056
	**p**	0.415	0.120	0.138	0.250
**OHIP-8**	**r**	0.013	0.022	0.018	0.029
	**p**	0.793	0.650	0.716	0.546
**OHIP-9**	**r**	0.017	0.004	-0.001	0.002
	**p**	0.734	0.942	0.981	0.967
**OHIP-10**	**r**	0.009	0.008	-0.022	0.040
	**p**	0.847	0.865	0.659	0.407
**OHIP-11**	**r**	-0.039	-0.028	-0.047	-0.036
	**p**	0.427	0.572	0.340	0.460
**OHIP-12**	**r**	0.039	0.014	0.018	0.026
	**p**	0.424	0.780	0.710	0.591
**OHIP-13**	**r**	0.050	0.058	0.078	0.026
	**p**	0.302	0.231	0.107	0.596
**OHIP-14**	**r**	-0.035	0.009	-0.021	-0.051
	**p**	0.474	0.848	0.663	0.294

Supplementary Table S8. Full comparisons across study-specific clinical periodontal status categories: demographic variables.

		**Clinical periodontal health** *n* = 204		**Gingivitis-compatible clinical status** *n* = 179		**Periodontitis-compatible clinical status** *n* = 40		
		**N**	**%**	**N**	**%**	**N**	**%**	**p**
Sex	Male	75	36.8%	50	27.9%	9	22.5%	0.081
	Female	129	63.2%	129	72.1%	31	77.5%	
Age	30–45	137	67.2%	105	58.7%	32	80.0%	0.106
	46–50	31	15.2%	33	18.4%	2	5.0%	
	51–60	23	11.3%	32	17.9%	5	12.5%	
	>60	13	6.4%	9	5.0%	1	2.5%	
Education	Primary School	19	9.3%	5	2.8%	0	0.0%	**<0.001**
	High School	59	28.9%	39	21.8%	4	10.0%	
	Undergraduate	79	38.7%	102	57.0%	25	62.5%	
	Master's	37	18.1%	26	14.5%	8	20.0%	
	PhD	10	4.9%	7	3.9%	3	7.5%	

Supplementary Table S9. Full comparisons across study-specific clinical periodontal status categories: awareness and behavior variables.

		**Clinical periodontal health** *n* = 204		**Gingivitis-compatible clinical status** *n* = 179		**Periodontitis-compatible clinical status** *n* = 40		
		**N**	**%**	**N**	**%**	**N**	**%**	**p**
Gingival bleeding	Yes	139	68.1%	138	77.1%	32	80.0%	0.091
	No	65	31.9%	41	22.9%	8	20.0%	
Gingival inflammation/infection	Yes	97	47.5%	70	39.1%	16	40.0%	0.232
	No	107	52.5%	109	60.9%	24	60.0%	
Gingival pain	Yes	107	52.5%	91	50.8%	26	65.0%	0.262
	No	97	47.5%	88	49.2%	14	35.0%	
Gingival swelling	Yes	76	37.3%	89	49.7%	24	60.0%	**0.006**
	No	128	62.7%	90	50.3%	16	40.0%	
Gingival itching	Yes	17	8.3%	13	7.3%	3	7.5%	0.963
	No	187	91.7%	166	92.7%	37	92.5%	
Cold/hot tooth sensitivity	Yes	69	33.8%	76	42.5%	14	35.0%	0.205
	No	135	66.2%	103	57.5%	26	65.0%	
Perceived gingival health	Yes	89	43.8%	86	48.3%	24	60.0%	0.079
	No	29	14.3%	32	18.0%	8	20.0%	
	Not sure	85	41.9%	60	33.7%	8	20.0%	
Toothbrushing frequency	Once a day	52	25.5%	48	27.0%	4	10.0%	0.135
	Twice a day	129	63.2%	111	62.4%	32	80.0%	
	3 times a day or more	10	4.9%	9	5.1%	4	10.0%	
	Sometimes	11	5.4%	10	5.6%	0	0.0%	
	Never	2	1.0%	0	0.0%	0	0.0%	
Awareness that gingival bleeding may indicate disease	Yes	133	65.2%	127	71.8%	27	67.5%	0.468
	No	40	19.6%	23	13.0%	8	20.0%	
	Don’t know	31	15.2%	27	15.3%	5	12.5%	
Halitosis	Yes	22	10.8%	31	17.5%	5	12.5%	0.160
	No	107	52.5%	72	40.7%	19	47.5%	
	Sometimes	75	36.8%	74	41.8%	16	40.0%	
Gingival bleeding during toothbrushing	Yes	52	25.5%	38	21.5%	9	22.5%	0.814
	No	55	27.0%	44	24.9%	11	27.5%	
	Sometimes	97	47.5%	95	53.7%	20	50.0%	
Regular dental visits	Yes	92	45.1%	71	39.9%	22	55.0%	0.206
	No	112	54.9%	107	60.1%	18	45.0%	
	Yes	139	68.1%	143	80.3%	35	87.5%	**0.004**

Previous professional dental scaling	No	65	31.9%	35	19.7%	5	12.5%	
Smoking status	5 cigarettes/day	14	6.9%	10	5.6%	4	10.0%	0.766
	10 cigarettes/day	19	9.3%	20	11.2%	3	7.5%	
	≥1 pack/day	38	18.6%	29	16.3%	4	10.0%	
	Non-smoker	133	65.2%	119	66.9%	29	72.5%	
Use of dental floss or interdental brushes	Yes	66	32.5%	68	38.2%	17	42.5%	0.339
	No	137	67.5%	110	61.8%	23	57.5%	
Awareness that professional dental scaling may help prevent periodontal diseases	Yes	107	52.5%	93	52.2%	27	67.5%	0.499
	No	40	19.6%	38	21.3%	6	15.0%	
	Don't know	57	27.9%	47	26.4%	7	17.5%	
Awareness of the association between periodontal diseases and cardiovascular disease/diabetes mellitus	Yes	95	46.8%	87	49.4%	24	60.0%	0.280
	No	69	34.0%	59	33.5%	7	17.5%	
	Not sure	39	19.2%	30	17.0%	9	22.5%	
Perceived need for periodontal treatment	Yes	177	87.2%	159	89.8%	35	87.5%	0.500
	No	6	3.0%	2	1.1%	2	5.0%	
	Not sure	20	9.9%	16	9.0%	3	7.5%	

Percentages were calculated using the number of valid responses for each variable; denominators may vary because of item-level missing responses.

The first six variables represent responses to the multiple-response question asking what first comes to mind when participants hear the term "gingival disease"; they do not represent current self-reported symptoms.

Supplementary Table S10. Full comparisons across study-specific clinical periodontal status categories: OHIP-14 item scores.

**Mean ± SD<br>Med. (Min.-Max.)**	**Clinical periodontal health<br>*n* = 204**	**Gingivitis-compatible clinical status<br>*n* = 179**	**Periodontitis-compatible clinical status<br>*n* = 40**	**p**
OHIP-1	1.6±1.01<br>1- (1-5)	1.62±0.91<br>1- (1-5)	1.53±0.82<br>1- (1-4)	0.650
OHIP-2	1.48±0.85<br>1- (1-5)	1.55±0.79<br>1- (1-5)	1.53±0.93<br>1- (1-5)	0.297
OHIP-3	2.34±0.99<br>2- (1-5)	2.44±0.94<br>2- (1-5)	2.45±0.99<br>3- (1-5)	0.529
OHIP-4	2±0.95<br>2- (1-5)	2.17±1.01<br>2- (1-5)	2.15±0.92<br>2- (1-4)	0.229
OHIP-5	2.86±1.34<br>3- (1-5)	3.06±1.2<br>3- (1-5)	3.15±1.39<br>3.5- (1-5)	0.256
OHIP-6	2.12±1.01[a]<br>2- (1-5)	2.4±0.96[a]<br>2- (1-5)	2.2±1.22<br>2- (1-5)	**0.018**
OHIP-7	1.56±0.89<br>1- (1-5)	1.69±0.87<br>1- (1-5)	1.65±1<br>1- (1-4)	0.094
OHIP-8	1.77±0.95<br>1- (1-5)	1.85±0.88<br>2- (1-5)	1.7±0.82<br>1.5- (1-4)	0.379
OHIP-9	2.14±1.03<br>2- (1-5)	2.2±0.99<br>2- (1-5)	2.1±1.1<br>2- (1-5)	0.631
OHIP-10	1.68±1<br>1- (1-5)	1.75±1.03<br>1- (1-5)	1.7±0.85<br>1- (1-4)	0.705
OHIP-11	1.54±0.81<br>1- (1-4)	1.46±0.79<br>1- (1-5)	1.68±0.97<br>1- (1-4)	0.405
OHIP-12	1.61±0.86<br>1- (1-5)	1.66±0.84<br>1- (1-5)	1.68±0.86<br>1- (1-4)	0.559
OHIP-13	1.51±0.85<br>1- (1-5)	1.62±0.88<br>1- (1-5)	1.63±0.98<br>1- (1-5)	0.267
OHIP-14	1.38±0.72<br>1- (1-4)	1.39±0.8<br>1- (1-5)	1.35±0.8<br>1- (1-5)	0.871

Data are presented as mean ± SD and median (min–max). The Kruskal–Wallis test was used for overall group comparisons. Different superscript letters within the same OHIP-14 item indicate statistically significant pairwise differences identified by Bonferroni-adjusted post-hoc comparisons.

Supplementary Table S11. Full comparisons between periodontal awareness/self-reported symptoms/oral-health behaviors and clinical periodontal indices at T0.

T0		GINGIVAL INDEX	BLEEDING ON PROBING %	PLAQUE INDEX	RAMFJORD PERIODONTAL INDEX
Gingival bleeding	Yes	1.09±1.01<br>1- (0-3)	6.76±6.81<br>5- (0-23)	1.35±1.09<br>1- (0-3)	1.58±1.69<br>1- (0-6)
	No	0.83±0.97<br>0.5- (0-3)	4.66±5.93<br>2- (0-23)	1.15±1.02<br>1- (0-3)	1.27±1.62<br>0- (0-6)
	**p[1]**	**0.019**	**0.005**	0.095	0.061
Gingival inflammation/infection	Yes	0.95±0.99<br>1- (0-3)	5.65±6.59<br>3- (0-23)	1.27±1.08<br>1- (0-3)	1.37±1.68<br>1- (0-6)
	No	1.08±1.02<br>1- (0-3)	6.61±6.66<br>5- (0-23)	1.32±1.08<br>1- (0-3)	1.6±1.67<br>1- (0-6)
	**p[1]**	0.187	0.076	0.598	0.086
Gingival pain	Yes	1.08±1.05<br>1- (0-3)	6.66±6.99<br>4- (0-23)	1.37±1.09<br>1- (0-3)	1.57±1.76<br>1- (0-6)
	No	0.95±0.95<br>1- (0-3)	5.68±6.21<br>4- (0-23)	1.22±1.06<br>1- (0-3)	1.42±1.58<br>1- (0-6)
	**p[1]**	0.256	0.249	0.176	0.488
Gingival swelling	Yes	1.22±1.05<br>1- (0-3)	7.2±7.03<br>6- (0-23)	1.48±1.06<br>2- (0-3)	1.76±1.74<br>1- (0-6)
	No	0.85±0.94<br>1- (0-3)	5.38±6.21<br>4- (0-22)	1.15±1.07<br>1- (0-3)	1.29±1.6<br>0.5- (0-6)
	**p[1]**	**<0.001**	**0.012**	**0.001**	**0.002**
Gingival itching	Yes	0.85±0.91<br>1- (0-3)	4.45±5.64<br>3- (0-23)	1.15±1.03<br>1- (0-3)	1.3±1.67<br>1- (0-6)
	No	1.03±1.01<br>1- (0-3)	6.34±6.71<br>4- (0-23)	1.31±1.08<br>1- (0-3)	1.52±1.68<br>1- (0-6)
	**p[1]**	0.357	0.164	0.424	0.474
Cold/hot tooth sensitivity	Yes	1.11±1.01<br>1- (0-3)	6.96±6.87<br>5- (0-23)	1.4±1.11<br>1- (0-3)	1.56±1.66<br>1- (0-6)
	No	0.97±1<br>1- (0-3)	5.74±6.47<br>3- (0-23)	1.23±1.06<br>1- (0-3)	1.46±1.69<br>1- (0-6)
	**p[1]**	0.149	0.061	0.135	0.367
Perceived gingival health	Yes	1.07±1.01<br>1- (0-3)	6.63±6.8<br>5- (0-23)	1.34±1.09<br>1- (0-3)	1.62±1.76<br>1- (0-6)
	No	1.17±1.01<br>1- (0-3)	7.33±7.24<br>7- (0-23)	1.52±1.11[a]<br>2- (0-3)	1.81±1.73[a]<br>2- (0-6)
	Not sure	0.89±0.99<br>1- (0-3)	5.14±6.05<br>4- (0-21)	1.14±1.03[a]<br>1- (0-3)	1.2±1.51[a]<br>1- (0-6)

	**p²**	0.087	0.065	**0.039**	**0.025**
Toothbrushing frequency	Once a day	0.97±0.96<br>1- (0-3)	6.46±6.67<br>4- (0-23)	1.3±0.95<br>1- (0-3)	1.32±1.52<br>1- (0-6)
	Twice a day	1.04±1.02<br>1- (0-3)	6.13±6.55<br>5- (0-23)	1.28±1.11<br>1- (0-3)	1.58±1.76<br>1- (0-6)
	3 times a day or more	1.17±1.11<br>1- (0-3)	7.39±8.09<br>6- (0-23)	1.57±1.24<br>2- (0-3)	1.83±1.83<br>1- (0-5)
	Sometimes	0.81±0.93<br>1- (0-3)	4.9±6.37<br>0- (0-17)	1.33±1.02<br>1- (0-3)	1.1±1.14<br>1- (0-3)
	Never	0±0<br>0- (0-0)	0±0<br>0- (0-0)	0±0<br>0- (0-0)	0±0<br>0- (0-0)
Awareness that gingival bleeding may indicate disease	Yes	1.06±1.02<br>1- (0-3)	6.35±6.78<br>4- (0-23)	1.36±1.1<br>1- (0-3)	1.52±1.68<br>1- (0-6)
	No	0.9±1<br>1- (0-3)	5.97±6.85<br>3- (0-22)	1.18±1.06<br>1- (0-3)	1.41±1.75<br>0- (0-6)
	Don’t know	0.95±0.97<br>1- (0-3)	5.68±5.86<br>5- (0-19)	1.14±0.98<br>1- (0-3)	1.48±1.64<br>1- (0-6)
	**p²**	0.405	0.859	0.266	0.687
Halitosis	Yes	1.33±1.05[a]<br>2- (0-3)	8.48±7.1[a]<br>10.5- (0-23)	1.55±1.1[a]<br>2- (0-3)	1.69±1.56<br>2- (0-5)
	No	0.9±0.96[a]<br>1- (0-3)	5.17±6.2[a]<br>3- (0-23)	1.16±1.03[a]<br>1- (0-3)	1.35±1.68<br>1- (0-6)
	Sometimes	1.04±1.03<br>1- (0-3)	6.61±6.82<br>5- (0-23)	1.37±1.12<br>1- (0-3)	1.6±1.72<br>1- (0-6)
	**p²**	**0.021**	**0.005**	**0.033**	0.100
Gingival bleeding during toothbrushing	Yes	0.97±1.03<br>1- (0-3)	6.17±6.88<br>4- (0-23)	1.2±1.06<br>1- (0-3)	1.39±1.65<br>1- (0-6)
	No	0.93±0.99<br>1- (0-3)	5.92±6.52<br>4- (0-23)	1.25±1.02<br>1- (0-3)	1.46±1.78<br>1- (0-6)
	Sometimes	1.08±1<br>1- (0-3)	6.34±6.64<br>5- (0-23)	1.37±1.12<br>1- (0-3)	1.56±1.64<br>1- (0-6)
	**p²**	0.320	0.846	0.427	0.528
Regular dental visits	Yes	1.01±1.01<br>1- (0-3)	6.14±6.74<br>4- (0-23)	1.32±1.11<br>1- (0-3)	1.52±1.8<br>1- (0-6)
	No	1.03±1.01<br>1- (0-3)	6.23±6.58<br>4- (0-23)	1.28±1.05<br>1- (0-3)	1.48±1.58<br>1- (0-6)
	**p¹**	0.833	0.959	0.763	0.680
Previous professional dental scaling	Yes	1.1±1.01<br>1- (0-3)	6.58±6.75<br>5- (0-23)	1.4±1.1<br>1- (0-3)	1.61±1.72<br>1- (0-6)
	No	0.77±0.96<br>0- (0-3)	5.01±6.22<br>0- (0-21)	0.98±0.96<br>1- (0-3)	1.17±1.52<br>0- (0-6)
	**p¹**	**0.003**	**0.029**	**0.001**	**0.022**

Smoking status	5 cigarettes/day	1.18±1.09<br>1- (0-3)	7.21±7.8<br>4- (0-23)	1.39±1.2<br>1- (0-3)	1.75±1.9<br>1- (0-6)
	10 cigarettes/day	0.98±1<br>1- (0-3)	5.4±5.1<br>5.5- (0-19)	1.4±1.13<br>1- (0-3)	1.4±1.58<br>1- (0-5)
	≥1 pack/day	0.83±0.93<br>0- (0-3)	4.97±6.06<br>0- (0-17)	1.18±1.02<br>1- (0-3)	1.37±1.52<br>1- (0-5)
	Non-smoker	1.05±1.02<br>1- (0-3)	6.51±6.85<br>5- (0-23)	1.3±1.08<br>1- (0-3)	1.52±1.71<br>1- (0-6)
	**p²**	0.358	0.317	0.753	0.869
Use of dental floss or interdental brushes	Yes	1.07±1.02<br>1- (0-3)	6.68±6.67<br>5- (0-23)	1.4±1.13<br>1- (0-3)	1.64±1.71<br>1- (0-6)
	No	0.99±1<br>1- (0-3)	5.94±6.63<br>4- (0-23)	1.24±1.05<br>1- (0-3)	1.42±1.66<br>1- (0-6)
	**p¹**	0.419	0.180	0.155	0.156
Awareness that professional dental scaling may help prevent periodontal diseases	Yes	1.05±1.03<br>1- (0-3)	6.08±6.67<br>4- (0-23)	1.29±1.08<br>1- (0-3)	1.56±1.76<br>1- (0-6)
	No	0.94±0.94<br>1- (0-3)	6.15±6.64<br>4- (0-23)	1.31±1.08<br>1- (0-3)	1.44±1.58<br>1- (0-5)
	Don’t know	1.01±1.02<br>1- (0-3)	6.42±6.66<br>5- (0-23)	1.3±1.08<br>1- (0-3)	1.42±1.59<br>1- (0-6)
	**p²**	0.777	0.853	0.989	0.945
Awareness of the association between periodontal diseases and cardiovascular disease/diabetes mellitus	Yes	1.1±1.03<br>1- (0-3)	6.4±6.86<br>4.5- (0-23)	1.36±1.1<br>1- (0-3)	1.63±1.75<br>1- (0-6)
	No	0.9±0.95<br>1- (0-3)	5.87±6.24<br>4- (0-23)	1.21±1.05<br>1- (0-3)	1.26±1.49<br>1- (0-5)
	Not sure	0.99±1.04<br>1- (0-3)	6.21±6.78<br>4- (0-23)	1.28±1.09<br>1- (0-3)	1.55±1.79<br>1- (0-6)
	**p²**	0.234	0.951	0.464	0.202
Perceived need for periodontal treatment	Yes	1.06±1.02<br>1- (0-3)	6.42±6.72<br>5- (0-23)	1.32±1.09<br>1- (0-3)	1.51±1.68<br>1- (0-6)
	No	0.5±0.85<br>0- (0-2)	4.1±6.85<br>0- (0-19)	0.9±0.74<br>1- (0-2)	1.1±2.08<br>0- (0-5)
	Not sure	0.74±0.88<br>0- (0-3)	4.82±5.75<br>4- (0-21)	1.15±1.06<br>1- (0-3)	1.49±1.65<br>1- (0-5)
	**p²**	**0.049**	0.220	0.358	0.444

Data are presented as mean ± SD and median (min–max). The Mann–Whitney U test was used for two-category comparisons, and the Kruskal–Wallis test was used for comparisons involving more than two categories. Different superscript letters within the same column indicate statistically significant pairwise differences identified by Bonferroni-adjusted post-hoc comparisons.

Supplementary Table S12. Full comparisons between periodontal awareness/self-reported symptoms/oral-health behaviors and clinical periodontal indices at T3.

T3		GINGIVAL INDEX	BLEEDING ON PROBING %	PLAQUE INDEX	RAMFJORD PERIODONTAL INDEX
Gingival bleeding	Yes	0.48±0.65<br>0- (0-2)	5.76±5.73<br>5- (0-19)	0.64±0.79<br>0- (0-2)	0.96±1.35<br>0- (0-5)
	No	0.33±0.59<br>0- (0-2)	4.04±5.13<br>0- (0-19)	0.47±0.71<br>0- (0-2)	0.76±1.24<br>0- (0-5)
	**p[1]**	**0.023**	**0.005**	0.052	0.139
Gingival inflammation/ infection	Yes	0.4±0.62<br>0- (0-2)	4.77±5.45<br>3- (0-19)	0.58±0.77<br>0- (0-2)	0.83±1.32<br>0- (0-5)
	No	0.47±0.65<br>0- (0-2)	5.7±5.72<br>5- (0-19)	0.61±0.77<br>0- (0-2)	0.97±1.33<br>0- (0-5)
	**p[1]**	0.216	0.075	0.634	0.173
Gingival pain	Yes	0.5±0.68<br>0- (0-2)	5.67±5.96<br>4- (0-19)	0.64±0.79<br>0- (0-2)	0.96±1.43<br>0- (0-5)
	No	0.38±0.58<br>0- (0-2)	4.88±5.2<br>4- (0-18)	0.54±0.74<br>0- (0-2)	0.85±1.2<br>0- (0-5)
	**p[1]**	0.093	0.312	0.199	0.884
Gingival swelling	Yes	0.58±0.68<br>0- (0-2)	6.1±5.93<br>5- (0-19)	0.71±0.79<br>1- (0-2)	1.07±1.42<br>0- (0-5)
	No	0.33±0.57<br>0- (0-2)	4.65±5.28<br>3.5- (0-18)	0.5±0.74<br>0- (0-2)	0.78±1.23<br>0- (0-5)
	**p[1]**	**<0.001**	**0.016**	**0.004**	**0.020**
Gingival itching	Yes	0.27±0.57<br>0- (0-2)	3.91±4.84<br>3- (0-19)	0.45±0.75<br>0- (0-2)	0.76±1.35<br>0- (0-5)
	No	0.45±0.64<br>0- (0-2)	5.42±5.67<br>4- (0-19)	0.61±0.77<br>0- (0-2)	0.92±1.32<br>0- (0-5)
	**p[1]**	0.078	0.160	0.211	0.329
Cold/hot tooth sensitivity	Yes	0.48±0.65<br>0- (0-2)	6±5.84<br>5- (0-19)	0.68±0.81<br>0- (0-2)	0.92±1.34<br>0- (0-5)
	No	0.41±0.62<br>0- (0-2)	4.88±5.45<br>3- (0-19)	0.55±0.74<br>0- (0-2)	0.9±1.32<br>0- (0-5)
	**p[1]**	0.247	**0.048**	0.098	0.807
Perceived gingival health	Yes	0.46±0.64<br>0- (0-2)	5.64±5.74<br>5- (0-19)	0.63±0.79<br>0- (0-2)	1.03±1.41[a]<br>0- (0-5)
	No	0.52±0.66<br>0- (0-2)	6.3±6.15<br>6- (0-19)	0.77±0.81[a]<br>1- (0-2)	1.14±1.39[b]<br>1- (0-5)
	Not sure	0.37±0.62<br>0- (0-2)	4.41±5.12<br>4- (0-17)	0.48±0.72[a]<br>0- (0-2)	0.65±1.14[a,b]<br>0- (0-5)

	**p[2]**	0.140	0.056	**0.021**	**0.007**
Toothbrushing frequency	Once a day	0.4±0.57<br>0- (0-2)	5.48±5.67<br>4- (0-18)	0.53±0.7<br>0- (0-2)	0.77±1.11<br>0- (0-5)
	Twice a day	0.45±0.66<br>0- (0-2)	5.25±5.53<br>4.5- (0-19)	0.61±0.78<br>0- (0-2)	0.99±1.41<br>0- (0-5)
	3 times a day or more	0.61±0.72<br>0- (0-2)	6.26±6.75<br>5- (0-19)	0.87±0.87<br>1- (0-2)	1.13±1.55<br>0- (0-4)
	Sometimes	0.29±0.56<br>0- (0-2)	4.29±5.5<br>0- (0-15)	0.52±0.81<br>0- (0-2)	0.48±0.81<br>0- (0-2)
	Never	0±0<br>0- (0-0)	0±0<br>0- (0-0)	0±0<br>0- (0-0)	0±0<br>0- (0-0)
Awareness that gingival bleeding may indicate disease	Yes	0.46±0.66<br>0- (0-2)	5.41±5.7<br>4- (0-19)	0.64±0.81<br>0- (0-2)	0.92±1.32<br>0- (0-5)
	No	0.39±0.6<br>0- (0-2)	5.13±5.94<br>3- (0-18)	0.52±0.73<br>0- (0-2)	0.92±1.37<br>0- (0-5)
	Don't know	0.41±0.56<br>0- (0-2)	4.9±5<br>5- (0-17)	0.48±0.62<br>0- (0-2)	0.87±1.3<br>0- (0-5)
	**p[2]**	0.856	0.820	0.384	0.936
Halitosis	Yes	0.66±0.66[a]<br>1- (0-2)	7.19±5.9[a]<br>9- (0-19)	0.81±0.76[a]<br>1- (0-2)	1.02±1.24<br>1- (0-4)
	No	0.36±0.6[a]<br>0- (0-2)	4.4±5.22[a]<br>2.5- (0-18)	0.48±0.72[a]<br>0- (0-2)	0.83±1.31<br>0- (0-5)
	Sometimes	0.46±0.65<br>0- (0-2)	5.69±5.82<br>5- (0-19)	0.65±0.82<br>0- (0-2)	0.96±1.37<br>0- (0-5)
	**p[2]**	**0.002**	**0.004**	**0.005**	0.216
Gingival bleeding during toothbrushing	Yes	0.44±0.64<br>0- (0-2)	5.24±5.72<br>4- (0-19)	0.55±0.72<br>0- (0-2)	0.86±1.29<br>0- (0-5)
	No	0.39±0.61<br>0- (0-2)	5.11±5.58<br>4- (0-19)	0.52±0.75<br>0- (0-2)	0.91±1.42<br>0- (0-5)
	Sometimes	0.46±0.65<br>0- (0-2)	5.41±5.64<br>5- (0-19)	0.66±0.8<br>0- (0-2)	0.93±1.3<br>0- (0-5)
	**p[2]**	0.643	0.889	0.245	0.756
Regular dental visits	Yes	0.43±0.64<br>0- (0-2)	5.32±5.85<br>4- (0-19)	0.63±0.79<br>0- (0-2)	0.98±1.44<br>0- (0-5)
	No	0.44±0.63<br>0- (0-2)	5.27±5.45<br>4- (0-19)	0.57±0.75<br>0- (0-2)	0.86±1.23<br>0- (0-5)
	**p[1]**	0.792	0.999	0.410	0.737
Previous professional dental scaling	Yes	0.47±0.65<br>0- (0-2)	5.62±5.74<br>5- (0-19)	0.67±0.8<br>0- (0-2)	0.98±1.37<br>0- (0-5)
	No	0.33±0.57<br>0- (0-2)	4.28±5.14<br>0- (0-17)	0.37±0.62<br>0- (0-2)	0.7±1.14<br>0- (0-5)
	**p[1]**	0.054	**0.036**	**0.001**	0.069

Smoking status	5 cigarettes/day	0.54±0.74<br>0- (0-2)	6.25±6.62<br>4- (0-19)	0.71±0.85<br>0- (0-2)	1.07±1.56<br>0- (0-5)
	10 cigarettes/day	0.4±0.63<br>0- (0-2)	4.67±4.44<br>5- (0-17)	0.67±0.85<br>0- (0-2)	0.83±1.19<br>0- (0-4)
	≥1 pack/day	0.34±0.51<br>0- (0-2)	4.27±5.21<br>0- (0-15)	0.51±0.69<br>0- (0-2)	0.79±1.15<br>0- (0-4)
	Non-smoker	0.46±0.65<br>0- (0-2)	5.54±5.76<br>4- (0-19)	0.59±0.77<br>0- (0-2)	0.94±1.36<br>0- (0-5)
	**p[2]**	0.657	0.323	0.734	0.956
Use of dental floss or interdental brushes	Yes	0.46±0.67<br>0- (0-2)	5.75±5.61<br>5- (0-19)	0.68±0.83<br>0- (0-2)	1.01±1.39<br>0- (0-5)
	No	0.43±0.62<br>0- (0-2)	5.05±5.63<br>4- (0-19)	0.55±0.73<br>0- (0-2)	0.86±1.29<br>0- (0-5)
	**p[1]**	0.890	0.145	0.149	0.233
Awareness that professional dental scaling may help prevent periodontal diseases	Yes	0.46±0.67<br>0- (0-2)	5.24±5.7<br>4- (0-19)	0.59±0.77<br>0- (0-2)	0.96±1.41<br>0- (0-5)
	No	0.37±0.55<br>0- (0-2)	5.3±5.62<br>4- (0-19)	0.6±0.78<br>0- (0-2)	0.87±1.2<br>0- (0-4)
	Don't know	0.45±0.63<br>0- (0-2)	5.39±5.52<br>5- (0-18)	0.59±0.77<br>0- (0-2)	0.85±1.24<br>0- (0-5)
	**p[2]**	0.698	0.924	0.999	0.965
Awareness of the association between periodontal diseases and cardiovascular disease/diabetes mellitus	Yes	0.49±0.68<br>0- (0-2)	5.49±5.85<br>4- (0-19)	0.65±0.79<br>0- (0-2)	1.01±1.41<br>0- (0-5)
	No	0.36±0.55<br>0- (0-2)	4.94±5.15<br>4- (0-19)	0.52±0.74<br>0- (0-2)	0.72±1.09<br>0- (0-4)
	Not sure	0.44±0.66<br>0- (0-2)	5.4±5.79<br>4- (0-19)	0.59±0.78<br>0- (0-2)	0.97±1.46<br>0- (0-5)
	**p[2]**	0.322	0.866	0.281	0.291
Perceived need for periodontal treatment	Yes	0.47±0.65<br>0- (0-2)	5.46±5.68<br>4- (0-19)	0.62±0.78<br>0- (0-2)	0.92±1.33<br>0- (0-5)
	No	0.2±0.42<br>0- (0-1)	3.7±6.06<br>0- (0-17)	0.2±0.42<br>0- (0-1)	0.8±1.69<br>0- (0-4)
	Not sure	0.26±0.5<br>0- (0-2)	4.31±4.93<br>4- (0-17)	0.49±0.76<br>0- (0-2)	0.87±1.24<br>0- (0-4)
	**p[2]**	0.078	0.299	0.146	0.613

Data are presented as mean ± SD and median (min–max). The Mann–Whitney U test was used for two-category comparisons, and the Kruskal–Wallis test was used for comparisons involving more than two categories. Different superscript letters within the same column indicate statistically significant pairwise differences identified by Bonferroni-adjusted post-hoc comparisons.
